# Machine learning guided process optimization and sustainable valorization of coconut biochar filled PLA biocomposites

**DOI:** 10.1038/s41598-025-19791-0

**Published:** 2025-10-05

**Authors:** Harishbabu Sundarasetty, Borhen Louhichi, Nashmi H. Alrasheedi, Santosh Kumar Sahu, It Ee Lee, Qamar Wali

**Affiliations:** 1 School of Mechanical Engineering, VIT-AP University, Besides A.P. Secretariat, Amaravati, 522237 Andhra Pradesh India; 2 Engineering Sciences Research Center (ESRC), Deanship of Scientific Research, Imam Mohammad Ibn Saud Islamic University (IMSIU), Riyadh, 11432 Saudi Arabia; 3 Department of Mechanical Engineering, College of Engineering, Imam Mohammad Ibn Saud Islamic University (IMSIU), Riyadh, 11432 Saudi Arabia; 4 Faculty of Artificial Intelligence and Engineering, Multimedia University, 63100 Cyberjaya, Malaysia; 5 Centre for Smart Systems and Automation, COE for Robotics and Sensing Technologies, Multimedia University, 63100 Cyberjaya, Malaysia

**Keywords:** Coconut shell biochar, ANOVA, Gradient boosting, XG-Boosting, Regression models, Engineering, Materials science

## Abstract

This study focuses on the valorization of coconut shell biochar (CCB) as a sustainable reinforcement in polylactic acid (PLA) biocomposites, targeting enhanced mechanical performance. PLA/CCB composites were fabricated by varying injection molding parameters at three levels: composition (Pure, 5 wt%, 10 wt%), injection temperature (135 °C, 145 °C, 155 °C), injection speed (50 mm/s, 60 mm/s, 70 mm/s), and injection pressure (30 bar, 40 bar, 50 bar). A Taguchi L27 orthogonal array was employed to systematically assess the effects of these parameters on tensile strength, Young’s modulus, and hardness. ANOVA results indicated that composition and injection temperature were the most influential factors, contributing 50.42% and 42.67% to tensile strength, and 38.58% and 20.14% to Young’s modulus, respectively. For hardness, composition dominated with a 78.3% contribution. To predict the mechanical responses, five machine learning models, including Linear Regression, Support Vector Regression (SVR), Random Forest Regression (RFR), Gradient Boosting, and Extreme Gradient Boosting (XGBoost), were implemented. Gradient Boosting and XGBoost exhibited superior predictive accuracy, with R^2^ values of 98.77% for tensile strength, 96.28% for Young’s modulus, and 96.45% for hardness. The integration of Taguchi design, ANOVA-based analysis, and advanced machine learning techniques offers a robust framework for optimizing process parameters and valorizing CCB as a high-performance, eco-friendly reinforcement in biodegradable biocomposites.

## Introduction

The growing concerns over environmental pollution and plastic waste have accelerated the search for sustainable alternatives to polymers^1,2^. Polylactic acid (PLA), a biodegradable thermoplastic derived from renewable resources such as corn starch and sugarcane, has emerged as a promising material owing to its environmental stability, compostability, and low carbon footprint^3,4^. PLA finds extensive applications in packaging, biomedical devices, and agricultural products due to its biocompatibility and favorable mechanical properties. In recent years, research has increasingly focused on enhancing the performance of PLA by incorporating various natural and organic fillers, thereby expanding its potential for high-performance, eco-friendly composite materials^5,6^. The following literature review summarizes key findings from recent research on PLA composites.

Joe et al.^7^ reported that Poly(lactic acid) PLA matrix reinforced with 1–3 wt% biochar derived from banana and orange peels exhibited a ~ 30% increase in tensile strength and a doubling of Young’s modulus from 194.33 MPa to 388.31 MPa. However, elongation at break decreased from 14.18 mm to 7.24 mm. SEM analysis revealed smooth fracture surfaces with minor agglomeration at higher filler loadings. Similarly, Vengadesan et al.^8^ incorporated 5–10 wt% rice husk and biocarbon fillers into PLA, achieving a tensile strength of 61.2 MPa (+ 105.6%), flexural strength of 135.8 MPa (+ 104.8%), and impact strength of 5.25 kJ/m^2^ at 10% filler content. Hardness increased to 80 Shore D, while ductility declined with higher filler levels. Papadopoulou et al.^9^ added 1–5 wt% biochar from softwood pellets into PLA via in situ polymerization. A 1 wt% addition raised tensile strength from ~ 30 MPa to ~ 32 MPa, but higher contents of 2.5–5 wt% resulted in brittleness and reduced strength to ~ 15–16 MPa. Yang et al.^10^ reported on PLA reinforced with pulp fiber to form biocomposites. Tensile tests showed that pulp fiber improved the tensile moduli but reduced the tensile strengths compared to neat PLA. Gurusamy et al.^11^ reinforced Turkish hemp fiber composites with 5–15 wt% pistachio shell biochar. Tensile strength improved from ~ 40 MPa to 54 MPa, flexural strength from ~ 50 MPa to 70 MPa, and impact resistance from ~ 4 kJ/m^2^ to 5 kJ/m^2^. Water absorption also decreased by ~ 15%. Mahesh et al.^12^ developed polymer composites containing 0–60 wt% eggshell and marble powder. At 60 wt%, marble-based composites exhibited 17.21% higher hardness than eggshell-based ones. Conversely, 60 wt% eggshell composites displayed 43.56% higher tensile strength. A hybrid formulation (50% eggshell, 10% marble) improved flexural and impact strengths by 10.67% and 10.9%, respectively, compared to 60% eggshell-only composites, while reversing the ratio enhanced impact strength by 52.45%. Uddin et al.^13^ reviewed the application of AI/ML approaches for predicting and optimizing the mechanical properties of PLA-based and natural fiber polymer composites. While specific values were not reported, the review highlighted AI/ML’s potential to address design complexities in next-generation biocomposites. Mallegni et al.^14^ incorporated 5–15 wt% insect exoskeleton biomass into PBSA/PHB-HV bioplastics. Tensile strength remained consistent at around 20 MPa across the formulations. SEM images confirmed well-dispersed fillers with minimal agglomeration. Mosi^15^ optimized coconut fiber-reinforced composites prepared via fused filament fabrication. At 30 wt% fiber content, tensile strength increased from 35 MPa to 43.8 MPa, flexural modulus improved from 2.1 GPa to 2.7 GPa, and impact resistance rose by 18%. Chawraba et al.^16^ added 10–30 wt% rice husk to wood–plastic composites. At 20 wt%, tensile strength rose from 25 MPa to 30 MPa, flexural modulus from 2.0 GPa to 2.5 GPa, and impact resistance increased by 15%. SEM confirmed good filler dispersion and bonding. Xia et al.^17^ reinforced PLA/PCL biodegradable composites with 5–20 wt% biochar. At 15 wt%, tensile strength increased from 45 MPa to 58 MPa, flexural modulus from 1.8 GPa to 2.3 GPa, and elongation at break rose by 12%. SEM images indicated uniform dispersion and strong interfacial adhesion. Botta et al.^18^ added 1–5 wt% biochar derived from biomass pyrolysis into recycled PLA, achieving up to a 20% increase in modulus. Malińska et al.^19^ incorporated 5–15 wt% biochar into bioplastics for horticultural applications and at 10 wt%, tensile strength increased from 22 MPa to 28 MPa, and elongation at break improved from 8% to 12%. George et al.^20^ reinforced PLA/PBAT (80/20) blends with 1–10 wt% pinewood biochar. At 1 wt%, tensile strength increased by 53%. SEM analysis confirmed good filler dispersion and adhesion. Zouari et al.^21^ investigated PLA and PLA–30% hemp fiber composites with 0–20 wt% biochar. In PLA, 5 wt% biochar raised tensile modulus from 2418 MPa to 3334 MPa without compromising strength (~ 37 MPa), while higher content reduced performance. In PLA–HF composites, 5–10% biochar improved modulus from 5158 MPa to 5841 MPa and tensile strength from 44 MPa to 51 MPa. George et al.^22^ fabricated PLA/PBAT (80/20) composites with coconut shell biochar (CCB) at 1 wt% and 10 wt%. At 1 wt%, tensile strength rose from ~ 37 MPa to ~ 54 MPa (+ 45%) and modulus increased from ~ 1.8 GPa to ~ 2.12 GPa (+ 18%).

Several studies have investigated using agricultural and plant-based wastes as fillers in polylactic acid (PLA)-based composites to enhance their mechanical properties for eco-friendly and engineering applications. Materials such as rice husk, hemp fibers, pistachio shell biochar, coconut shell biochar (CCB), eggshell powder, and other biochars have been explored, with coconut shell-derived biochar (coco char) receiving particular attention due to its abundant availability, low cost, high fixed carbon content, biodegradability, and strong reinforcement potential. Alongside filler selection, processing methods critically influence composite performance. Injection moulding is widely used for PLA because of its precision and scalability, yet parameters such as temperature, speed, pressure, and filler loading strongly affect final properties, requiring systematic optimization. Taguchi design of experiments provides a resource-efficient approach to identify significant factors, while machine learning captures complex non-linear interactions for accurate prediction. Their integration ensures efficiency and robustness, while CCB further enhances environmental sustainability by valorizing agricultural waste into biodegradable composites.

## Materials and methods

### Materials

**Fig. 1:**
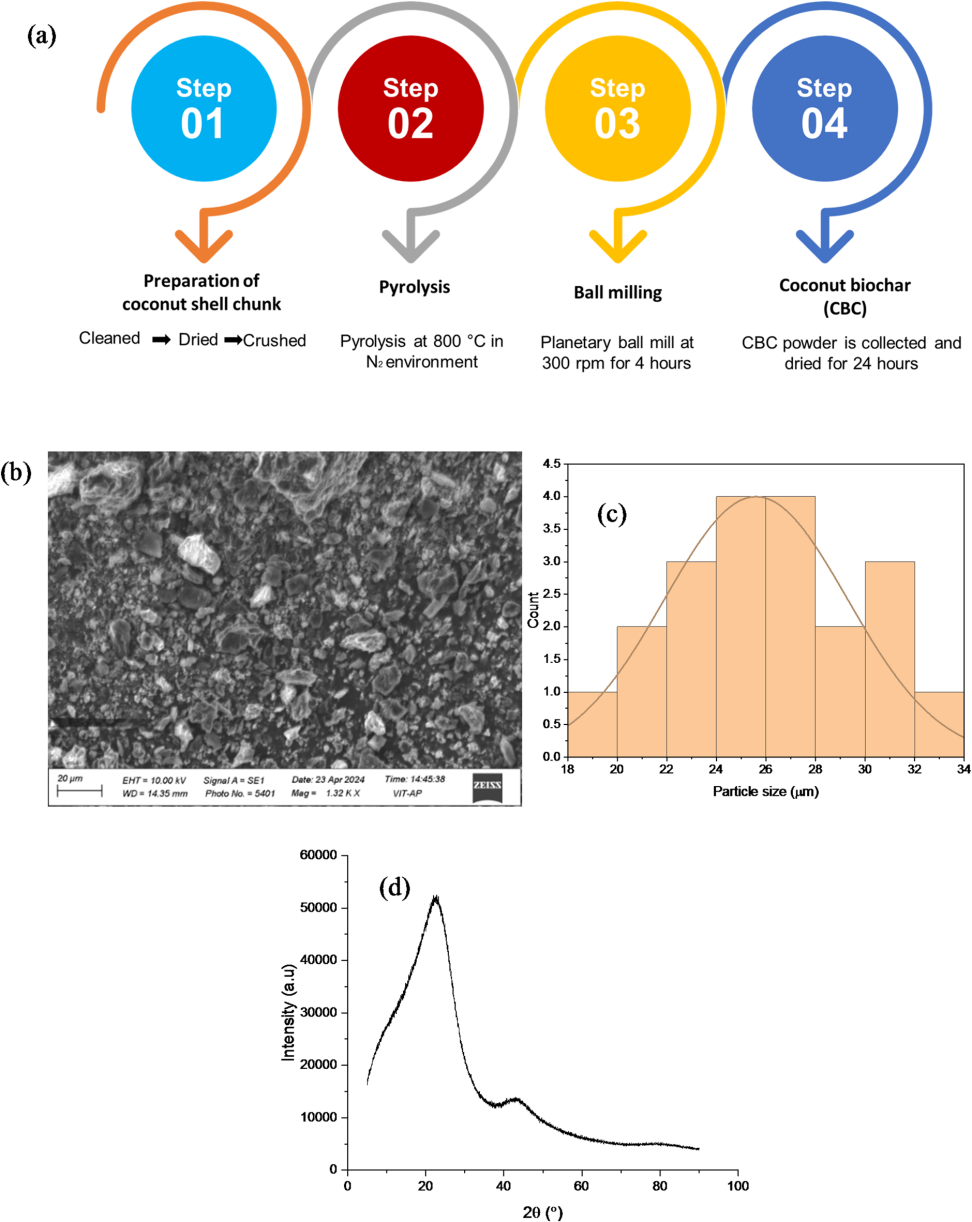
(**a**) Synthesis process; (**b**) SEM morphology; (**c**) Particle size distribution histogram; (**d**) XRD analysis CCB powder.

Polylactic acid (PLA) granules, with a density ranging from 1.20 to 1.30 g/cc and a melt flow index (MFI) specified in g/10 min, were sourced from Banka BioLoo Limited, India. Coconut shell biochar (CCB), used as the reinforcing filler, was prepared through the process illustrated in Fig. 1. Discarded coconut shells were first cleaned, dried, and pyrolyzed at 800 °C under a nitrogen (N₂) atmosphere, which creates an inert environment to prevent oxidation and combustion of the coconut shells during thermal decomposition of the shells into biochar. The resulting char was ground and subsequently ball-milled at 300 rpm for 4 h with a ball-to-powder weight ratio of 10:1. To remove any moisture content, the char was kept in an oven for 24 h^23^. The SEM analysis, as shown in Fig. 1b, is performed on the collected char to analyse the morphology of the CCB powder and noted irregular, porous particles. In contrast, particle size analysis (Fig. 1c) confirmed an average particle size of 25 μm. The XRD pattern of CCB powder displayed in Fig. 1d and noted a broad peak around 22°–24° (2θ), attributed to the (002) plane of disordered carbon, along with a weaker hump near 43°–45° (2θ), corresponding to partially ordered carbon structures. The absence of sharp crystalline peaks confirmed its predominantly amorphous carbon nature^24^.

### Taguchi experiment design

Before preparing the samples, a Taguchi Design of Experiments (DoE) approach was employed to evaluate the influence of multiple processing parameters while minimizing the total number of experimental trials. This study selected four key injection molding parameters — composition, injection temperature, injection speed, and injection pressure — each varied at three levels as detailed in Table 1^25^. An L_27_ orthogonal array was generated using Minitab 2021 software to systematically arrange the combinations of these parameters, as presented in Tables 2 and 3. This structured experimental design ensured a balanced analysis of variable effects while optimizing time, effort, and material resources^26,27^.

**Table 1:** Process parameters and levels for composite preparation.

Sl. no.	Factors	Units	Level 1	Level 2	Level 3
1	Composition	wt%	Pure	5	10
2	Temperature	°C	135	145	155
3	Injection speed	mm/s	50	60	70
4	Injection pressure	Bar	30	40	50

**Table 2:** Taguchi L_27_ orthogonal array with experimental values of tensile strength, young’s modulus, and corresponding S/N ratios.

Sl. no.	Composition	Injection temp.	Injection speed	Injection pressure	Tensile strength	Young’s modulus	S/*N* ratio (tensile strength)	S/*N* ratio (Young’s modulus)
1	Pure	135	50	30	17.4000	1230.01	24.8605	60.8016
2	Pure	135	50	30	17.6000	1159.14	24.8605	60.8016
3	Pure	135	50	30	17.5000	958.21	24.8605	60.8016
4	Pure	145	60	40	5.7300	820.53	14.9509	58.5081
5	Pure	145	60	40	5.4600	915.14	14.9509	58.5081
6	Pure	145	60	40	5.5950	803.13	14.9509	58.5081
7	Pure	155	70	50	1.2700	753.18	4.5123	58.0529
8	Pure	155	70	50	2.5000	693.88	4.5123	58.0529
9	Pure	155	70	50	1.8850	1080.00	4.5123	58.0529
10	5	135	60	50	58.5000	1006.49	35.1115	60.4710
11	5	135	60	50	55.5000	1018.16	35.1115	60.4710
12	5	135	60	50	57.0000	1162.57	35.1115	60.4710
13	5	145	70	30	39.2000	742.55	31.8098	56.9588
14	5	145	70	30	38.7000	651.66	31.8098	56.9588
15	5	145	70	30	38.9500	730.42	31.8098	56.9588
16	5	155	50	40	24.0000	553.43	26.8233	54.7709
17	5	155	50	40	20.2000	555.02	26.8233	54.7709
18	5	155	50	40	22.1000	535.33	26.8233	54.7709
19	10	135	70	40	54.6966	3561.48	35.2199	69.9311
20	10	135	70	40	60.9992	2652.43	35.2199	69.9311
21	10	135	70	40	57.8479	3453.98	35.2199	69.9311
22	10	145	50	50	38.7000	1637.39	29.7934	64.1668
23	10	145	50	50	25.7000	1433.38	29.7934	64.1668
24	10	145	50	50	32.2000	1858.16	29.7934	64.1668
25	10	155	60	30	17.8000	107.94	23.9027	40.6626
26	10	155	60	30	14.0000	105.84	23.9027	40.6626
27	10	155	60	30	15.9000	110.12	23.9027	40.6626

**Table 3:** Taguchi L_27_ orthogonal array with experimentally obtained Harness with S/N ratio.

Sl. no.	Composition	Temp	Injection speed	Injection pressure	Hardness	S/*N* ratio (hardness)
1	Pure	135	50	30	32.30	30.3730
2	Pure	135	50	30	32.80	30.3730
3	Pure	135	50	30	36.10	30.3730
4	Pure	145	60	40	35.90	30.8592
5	Pure	145	60	40	33.00	30.8592
6	Pure	145	60	40	42.60	30.8592
7	Pure	155	70	50	37.60	31.6778
8	Pure	155	70	50	35.80	31.6778
9	Pure	155	70	50	40.40	31.6778
10	5	135	60	50	37.20	31.5347
11	5	135	60	50	36.00	31.5347
12	5	135	60	50	39.20	31.5347
13	5	145	70	30	43.00	32.3131
14	5	145	70	30	41.90	32.3131
15	5	145	70	30	46.80	32.3131
16	5	155	50	40	36.10	32.2075
17	5	155	50	40	41.50	32.2075
18	5	155	50	40	56.20	32.2075
19	10	135	70	40	51.40	34.2085
20	10	135	70	40	47.50	34.2085
21	10	135	70	40	62.90	34.2085
22	10	145	50	50	60.90	35.6882
23	10	145	50	50	59.00	35.6882
24	10	145	50	50	77.00	35.6882
25	10	155	60	30	74.60	37.4242
26	10	155	60	30	71.70	37.4242
27	10	155	60	30	32.30	37.4242

### Sample Preparation

**Fig. 2:**
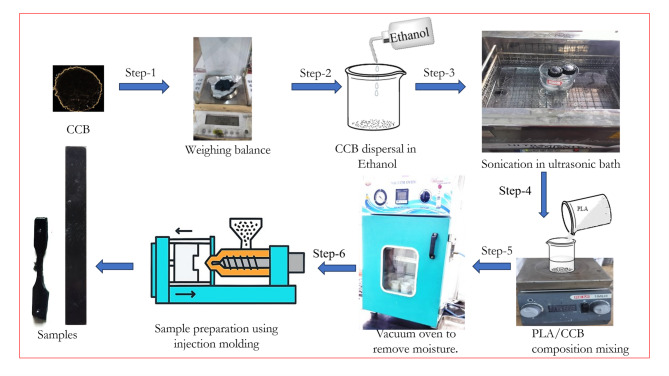
Systemic steps for sample preparation.

Composite and pure PLA samples were fabricated using polylactic acid (PLA) as the base polymer, reinforced with coco biochar (CCB), as illustrated in Fig. 2. The fabrication process began with precise weighing of PLA and CCB using a high-precision balance (Shimadzu ATX-224, Japan). To ensure uniform dispersion, CCB was first suspended in ethanol and subjected to ultrasonication. PLA granules were then gradually introduced into the CCB–ethanol suspension while continuously stirring on a magnetic hot plate to promote homogeneous mixing. The blended mixture was subsequently dried in a vacuum oven (GR-58, Nano Tec, India) at 70 °C for 24 h to remove ethanol and residual moisture. The dried materials were processed using a semi-automatic horizontal injection molding machine (Deesha Impex Pvt. Ltd., India). Test specimens were prepared as per ASTM standards with filler loadings of 5 wt% and 10 wt% in PLA, along with pure PLA. During molding, key processing parameters—composition, injection temperature, injection speed, and injection pressure—were systematically varied as shown in Table 1^27,28^.

### Mechanical testing

#### Tensile testing

The tensile properties of the injection-moulded specimens were assessed using a Universal Testing Machine (UTM, Model H10KL, Tinius Olsen India Pvt. Ltd., India) per ASTM D638 standards. Specimens, prepared based on the L27 orthogonal array design, were subjected to uniaxial tensile loading at a constant strain rate of 2 mm/min to determine their tensile strength and Young’s modulus. To ensure the accuracy and reproducibility of results, each test was conducted in triplicate, and the average values were reported.

##### Scanning electron microscope

Following the tensile tests, the fracture surfaces of the specimens were analyzed using a Scanning Electron Microscope (ZEISS EVO 10, Germany) to investigate the failure mechanisms and surface morphology. Before SEM examination, the fractured surfaces were sputter-coated with a thin layer of gold to enhance surface conductivity and were imaged under appropriate accelerating voltage conditions^29,30^.

#### Hardness testing

The Vickers hardness test is a widely used for evaluating a material’s resistance to plastic deformation, particularly suitable for polymers and their composites. This technique utilizes a diamond-shaped pyramidal indenter with a face angle of 136°, which is pressed into the material’s surface under a controlled load. The present study measured pure PLA and its composites’ hardness per ASTM E384 using a Micro Vickers Hardness Tester (MC-AT, Fine Spavy Associates & Engineers Pvt. Ltd., India). A micro-indentation load of 0.5 kg was applied to each specimen for a dwell time of 10 s^31–33^. Following the removal of the load, the diagonals of the resulting indentations were measured using an optical microscope, and the Hardness Value (HV) was calculated using Eq. (1)^34^. All measurements were conducted at room temperature, and the average of three indentations per sample was reported to ensure accuracy and consistency.

1 $$\:\text{H}\text{V}\hspace{0.17em}=\hspace{0.17em}2\frac{\text{P\:sin}\frac{\text{136}}{\text{2}}}{{\text{x}}^{\text{2}}}$$

Here, HV IS Vickers Hardness, P is the applied load in kgf, and x is the average length of the two diagonals.

### Static analysis

#### Taguchi analysis

The Taguchi method was applied during the sample preparation phase to investigate the influence of selected processing parameters on the mechanical properties of PLA and CCB-reinforced composites, including tensile strength, Young’s modulus, and hardness. Specimens were fabricated according to the combinations specified in the Taguchi L_27_ orthogonal array, allowing for the systematic variation of key parameters based on the predefined design of experiments (DOE). This approach provided an efficient and statistically reliable experimental framework that reduced the required trials while effectively capturing the interactions among critical factors affecting the mechanical performance. Each experimental condition was replicated to ensure the consistency and reliability of the results. The measured responses were subsequently analyzed using signal-to-noise (S/N) ratios, employing the “larger-is-better” criterion, as presented in Tables 2 and 3 and calculated using Eq. (2)^35^, to identify the optimal processing conditions for improved mechanical properties.

2 $$\:\frac{\text{S}}{\text{N}}\:\text{r}\text{a}\text{t}\text{i}\text{o}=-10{\text{log}}_{10}\left[\frac{1}{\text{k}}\sum\:_{\text{i}=1}^{\text{k}}\frac{1}{{{\text{C}}_{\text{i}}}^{2}}\right]$$

#### Analysis of variance (ANOVA)

In addition to the Taguchi signal-to-noise (S/N) ratio analysis, analysis of variance (ANOVA) was performed to statistically evaluate the significance and contribution of each processing parameter to the mechanical properties of PLA and CCB-reinforced composites. ANOVA identifies the factors that exert a statistically significant influence on output responses — including tensile strength, Young’s modulus, and hardness — by comparing the mean square values and corresponding F-ratios using Minitab 2021. The results offer valuable insight into each parameter’s relative impact, along with individual factors’ percentage contribution to the overall variation in mechanical performance. Parameters with p-values less than 0.05 were considered statistically significant at a 95% confidence level. This statistical evaluation complements the Taguchi method by reinforcing the reliability of the optimal processing conditions established through signal-to-noise ratio analysis^36,37^.

### Machine learning

Machine learning (ML) is a modern and efficient technique increasingly used to optimize the mechanical properties of materials, offering significant advantages over traditional trial-and-error methods. ML models predict and optimize outcomes derived from experiments while substantially reducing the required effort. By capturing complex, nonlinear relationships between processing parameters and mechanical responses, ML provides effective guidance for material design and process optimization. In this study, ML approaches were employed to optimize injection molding process parameters for PLA and CCB-reinforced composites. The dataset generated from the Taguchi experimental design was used to develop and evaluate regression models using Google Colab with Python libraries such as Scikit-learn, XG-Boost, Pandas, and NumPy. The specific ML algorithms applied are discussed in detail in the following section^38^. Model performance was assessed using standard evaluation metrics, including Mean Squared Error (MSE), Root Mean Squared Error (RMSE), Relative RMSE (RRMSE), Mean Absolute Error (MAE), Mean Absolute Percentage Error (MAPE), and the coefficient of determination (R^2^). These metrics were calculated using Eqs. (3)-(8)^39–41^.

3 $$\:{\text{R}}^{2}=1-\frac{\sum\:_{\text{i}=1}^{\text{k}}{{(\text{C}}_{\text{i}}-{\stackrel{-}{\text{C}}}_{\text{i}})}^{2}}{\sum\:_{\text{i}=1}^{\text{k}}{{(\text{C}}_{\text{i}}-\stackrel{-}{\text{C}})}^{2}}$$

4 $$\:\text{M}\text{S}\text{E}=\frac{1}{\text{k}}\sum\:_{\text{i}=1}^{\text{k}}{{(\text{C}}_{\text{i}}-{\stackrel{-}{\text{C}}}_{\text{i}})}^{2}$$

5 $$\:\text{R}\text{M}\text{S}\text{E}=\sqrt{\text{M}\text{S}\text{E}}=\:\sqrt{{{(\text{C}}_{\text{i}}-{\stackrel{-}{\text{C}}}_{\text{i}})}^{2}}$$

6 $$\:\text{R}\text{R}\text{M}\text{S}\text{E}=\frac{\text{R}\text{M}\text{S}\text{E}}{\stackrel{-}{\text{O}}}=\frac{\sqrt{{{(\text{C}}_{\text{i}}-{\stackrel{-}{\text{C}}}_{\text{i}})}^{2}}}{\stackrel{-}{\text{C}}}\:\times\:\:100$$

7 $$\:\text{M}\text{A}\text{E}=\:\frac{1}{\text{k}}\sum\:_{\text{i}=1}^{\text{k}}\left|{\text{C}}_{\text{i}}-{\stackrel{-}{\text{C}}}_{\text{i}}\right|$$

8 $$\:\text{M}\text{A}\text{P}\text{E}=\:\frac{1}{\text{k}}\sum\:_{\text{i}=1}^{\text{k}}\left|\frac{{\text{C}}_{\text{i}}-{\stackrel{-}{\text{C}}}_{\text{i}}}{{\text{C}}_{\text{i}}}\right|\:\times\:100$$

Here, k is the number of trials $\:{\mathbf{C}}_{\mathbf{i}}$and $\:\stackrel{-}{\mathbf{C}}$ are the actual values and the mean of the actual values, respectively, similarly $\:{\stackrel{-}{\mathbf{C}}}_{\mathbf{i}}$ is the predicted value.

#### Linear regression

Linear Regression (LR) is a fundamental supervised learning algorithm used to model the relationship between a dependent variable and one or more independent variables by fitting a linear equation to the observed data, as represented in Eqs. (9) and (10)^42^. In this study, LR was employed to predict the mechanical properties of PLA and its composites based on selected processing parameters. The algorithm works by minimizing the residual sum of squares between the actual and predicted values, providing a straightforward yet effective baseline model for comparing the performance of more advanced machine learning techniques.

9 $$\:\stackrel{-}{\text{C}}={\text{l}}_{0}+{\text{l}}_{1}{\text{R}}_{1}+{\text{l}}_{2}{\text{R}}_{2}+{\text{l}}_{3}{\text{R}}_{3}+{\text{l}}_{4}{\text{R}}_{4}$$

Here $\:{\text{R}}_{1},\:{\text{R}}_{2},\:{\text{R}}_{3},\:{\text{R}}_{4}$ are the composition, Temperature, injection speed and injection pressure, respectively. and $\:{\text{l}}_{0},\:{\text{l}}_{1}$, $\:{\text{l}}_{2},\:{\text{l}}_{3},\:\text{a}\text{n}\text{d}\:{\text{l}}_{4}\:$are the model coefficients.

Model coefficient estimation:

10 $$\:\stackrel{-}{\text{l}}={\left({\text{Q}}^{\text{T}}\text{Q}\right)}^{-1}{\text{Q}}^{\text{T}}\text{y}$$

Here, Q is the scaled input parameters matrix, and C is the scaled output parameters, such as tensile strength, Young’s modulus and hardness.

#### Support vector regression (SVR)

Support Vector Regression (SVR) is a machine learning algorithm derived from the principles of Support Vector Machines (SVM), designed to predict continuous outcomes. It works by identifying a function that approximates the data within a specified error margin (ε-insensitive zone) while keeping the model’s complexity as low as possible. In this study, SVR is utilized to predict the mechanical properties of PLA and its composites, employing kernel functions to capture both linear and nonlinear relationships between the input variables and output responses. Among these, the radial basis function (RBF) kernel is widely used for its flexibility and effectiveness in handling nonlinear data. The underlying concepts and formulations are presented in Eqs. (11), (12), and (13)^43^.

11 $$\:\text{F}\left(\text{Q}\right)={\text{w}}^{\text{T}}\text{q}+\text{b}$$

Here, $\:\text{Q}\:\text{i}\text{s}\:{\left[\text{C}\text{o}\text{m}\text{p}\text{o}\text{s}\text{i}\text{t}\text{i}\text{o}\text{n},\:\text{T}\text{e}\text{m}\text{p}\text{e}\text{r}\text{a}\text{t}\text{u}\text{r}\text{e},\:\text{I}\text{n}\text{j}\text{e}\text{c}\text{t}\text{i}\text{o}\text{n}\:\text{S}\text{p}\text{e}\text{e}\text{d},\:\text{I}\text{n}\text{j}\text{e}\text{c}\text{t}\text{i}\text{o}\text{n}\:\text{P}\text{r}\text{e}\text{s}\text{s}\text{u}\text{r}\text{e}\right]}^{\text{T}}\:$ is the input vector, $\:\text{w}$ is the weight vector, b is the bias term, and $\:{\text{w}}^{\text{T}}\text{q}$ is the dot product between $\:\text{w}$ and $\:\text{Q}$.

Epsilon-Insensitive Loss Function is:

12 $$\:\text{L}\left(\text{C},\:\text{f}\left(\text{Q}\right)\right)=\left\{\begin{array}{c}0\:\:\:\:\:\:\:\:\:\:\:\:\:\:\:\:\:\:\:\:\:\:\:\:\:\:\:\:\:\:\:if\:\left|\text{C}-\text{f}\left(\text{q}\right)\right|<{\text{\euro }} \\\:\left|\text{y}-\text{f}\left(\text{q}\right)\right|-{\text{\euro }} \:\:\:\:\:\:\:\:\:\:\:\:\:\:\:\:other\:wise\:\end{array}\:\:\right.$$

Here, y is the actual output variables (Tensile Strength, Young’s Modulus, and Hardness), $\:\text{f}\left(\text{Q}\right)$ is the predicted output function and ${\text{\euro }}$ is the Epsilon-insensitive margin (tolerance zone).

Objective optimization is:

13 $$\begin{gathered} \mathop {\hbox{min} }\limits_{{{\text{w}},{\text{~b}},{\text{~d}},{{\text{d}}^{\text{*}}}}} \left( {\frac{1}{2}{{\text{w}}^2}+{\text{C}}\mathop \sum \limits_{{{\text{i}}=1}}^{{\text{k}}} {{\text{d}}_{\text{i}}}+{{\text{d}}^{\text{*}}}_{{\text{i}}}} \right) \hfill \\ {\text{Subject to}} \hfill \\ {{\text{C}}_{\text{i}}} - {{\text{w}}^{\text{T}}}{{\text{q}}_{\text{i}}} - {\text{b}} \leqslant {\text{\euro }}+{{\text{d}}_{\text{i}}} \hfill \\ {{\text{w}}^{\text{T}}}{{\text{q}}_{\text{i}}}+{\text{b}} - {{\text{C}}_{\text{i}}} \leqslant {\text{\euro }}+{{\text{d}}^{\text{*}}}_{{\text{i}}} \hfill \\ {{\text{d}}_{\text{i}}},{{\text{d}}_{\text{i}}}^{{\text{*}}} \geqslant 0 \hfill \\ \end{gathered}$$

Here $\:{\text{d}}_{\text{I}},{{\text{d}}_{\text{i}}}^{\text{*}}$—Slack variables for positive and negative deviations beyond ${\text{\euro }}$, C—is a regularisation parameter controlling the penalty of errors, and k—is the Total number of data points.

#### Random forest regression (RFR)

Random Forest Regression (RFR) is a widely used machine learning algorithm for regression tasks, valued for its high accuracy, robustness, and ability to model complex relationships between input features and target variables. It is particularly effective at handling nonlinear data and reducing the risk of overfitting. As an ensemble learning technique, RFR improves predictive performance by combining the outputs of multiple decision trees. Each tree is trained on a bootstrapped subset of the training data and considers a random selection of input features at each split, which enhances the model’s robustness and generalization ability. The underlying methodology is represented by Eqs. (14) and (15)^44^.

Define a dataset by:

14 $$\:\text{D}=\left\{\right({\text{Q}}_{\text{i}},{\text{C}}_{\text{i}}\left)\right\}\genfrac{}{}{0pt}{}{\text{k}}{\text{i}=1}$$

Here $\:{\text{Q}}_{\text{I}}$ is [Composition, temperature, Injection Speed, Injection Pressure], and $\:{\text{C}}_{\text{I}}$ is [Tensile Strength, Young’s Modulus, Hardness].

For the given input factors P, the Random Forest Regression consists of k regression trees.

$\:{\text{f}}_{1}\left(\text{Q}\right)$, $\:{\text{f}}_{2}\left(\text{Q}\right),\:{\text{f}}_{3}\left(\text{Q}\right)$, …….$\:{\text{f}}_{\text{n}}\left(\text{Q}\right)$ predicts the output $\:\stackrel{-}{\text{C}}$ as the average of the individual tree predictions

15 $$\:\stackrel{-}{\text{C}}\left(\text{Q}\right)=\frac{1}{\text{K}}\sum\:_{\text{n}=1}^{\text{N}}{\text{f}}_{\text{n}}\left(\text{Q}\right)\:$$

Here $\:\stackrel{-}{\text{C}}\left(\text{Q}\right)$ is the predicted value for the input P, N is the total number of decision trees in the forest, and $\:{\text{f}}_{\text{n}}\left(\text{Q}\right)$ is the prediction from the $\:{\text{K}}^{\text{t}\text{h}}$ tree.

#### Gradient boosting

Gradient Boosting Regression (GBR) is an ensemble machine learning technique specifically designed for regression tasks involving complex and nonlinear relationships. It operates by minimizing a chosen loss function through gradient descent, sequentially building predictive models that correct the errors of preceding models. GBR combines multiple weak learners, typically shallow decision trees, into a strong, unified model, thereby improving predictive accuracy and reducing bias. Unlike Random Forests, which rely on bagging and train decision trees in parallel, GBR follows a boosting strategy, where models are trained sequentially with greater emphasis on the most difficult-to-predict samples. This approach makes GBR particularly well-suited for modeling the mechanical properties of composite materials influenced by multiple processing parameters. The mathematical framework of this method, including the additive model and gradient-based update process, is detailed in Equations (16) to (19)^45^.

Define a dataset by,

16 $$\:\text{D}=\left\{\right({\text{Q}}_{\text{i}},{\text{C}}_{\text{i}}\left)\right\}\genfrac{}{}{0pt}{}{\text{m}}{\text{i}=1}$$

Here $\:{\text{Q}}_{\text{i}}$ is [composition, temperature, injection speed, injection pressure], and $\:{\text{C}}_{\text{i}}$ is [tensile strength, Young’s modulus, hardness]

17 $$\:{\text{F}}_{\text{M}}\left(\text{Q}\right)=\sum\:_{\text{m}=1}^{\text{m}}{\text{R}}_{\text{m}}{\text{h}}_{\text{m}}\left(\text{Q}\right)$$

For the gradient descent update

18 $$\:{\text{F}}_{\text{m}}\left(\text{Q}\right)={\text{F}}_{\text{m}-1}\left(\text{Q}\right)+{\text{R}}_{\text{m}}{\text{h}}_{\text{m}}\left(\text{Q}\right)$$

Here $\:{\text{R}}_{\text{m}}$ is the learning rate or weight applied to each tree, M is the total number of iterations (trees), and $\:{\text{h}}_{\text{m}}\left(\text{Q}\right)$ is the $\:{\text{m}}^{\text{t}\text{h}}$weak learner (decision tree).

h_m_ is trained to fit the pseudo-residuals

19 $$\:{\text{h}}_{\text{m}}\approx\:\:{\text{r}}_{\text{i}}^{\text{m}}=\left[\frac{\partial\:\text{l}\left({\text{C}}_{\text{i}},{\text{F}}_{\text{m}-1}\left({\text{Q}}_{\text{i}}\right)\right)}{\partial\:{\text{F}}_{\text{m}-1}\left({\text{Q}}_{\text{i}}\right)}\right]\:$$

Here $\:{\text{r}}_{\text{i}}^{\text{m}}$ is the pseudo-residual for data point i at boosting iteration m, $\:{\text{C}}_{\text{i}}$is the actual target value, $\:{\text{F}}_{\text{m}-1}\left({\text{Q}}_{\text{i}}\right)$ is Prediction from the model after m − 1 iterations, and $\:\partial\:\text{l}\left({\text{C}}_{\text{i}},{\text{F}}_{\text{m}-1}\left({\text{Q}}_{\text{i}}\right)\right)$ is the loss function (e.g., mean squared error).

#### Extreme gradient boosting (XG-Boost)

Extreme Gradient Boosting (XG-Boost) is a highly effective and widely adopted machine learning algorithm in both academic research and industrial applications, recognized for its scalability, regularization features, and strong resistance to overfitting. Unlike conventional gradient boosting techniques, XG-Boost enhances model performance by incorporating both L1 and L2 regularization terms into its loss function, improving generalization and controlling model complexity. In this study, XG-Boost is applied to model and predict the mechanical properties — tensile strength, Young’s modulus, and hardness — of PLA/CCB composites, with composition, temperature, injection speed, and injection pressure serving as input variables. The algorithm builds an ensemble of regression trees by minimizing a regularized objective function, as described in Eqs. (20) and (21)^46,47^.

20 $$\:\text{L}\left(\text{t}\right)=\sum\:_{\text{i}=1}^{\text{n}}\left(\text{l}\right({\text{C}}_{\text{i}}{{\stackrel{-}{\text{C}}}_{\text{i}}}^{\text{t}})+\sum\:_{\text{m}=1}^{\text{t}}{\upphi\:}\left({\text{f}}_{\text{m}}\right)$$

21 $$\:{\upphi\:}\left(\text{f}\right)={\upbeta\:}\text{T}+\frac{1}{2}{\updelta\:}{\|{\upomega\:}\|}^{2}$$

Here $\:\text{l}$ is a differentiable loss function, $\:{\text{f}}_{\text{m}}\:$represents an individual regression tree at iteration $\:\text{m}$, $\:\text{T}$ is the number of leaves in the tree, $\:{\upomega\:}$ is the vector of scores on leaves, and $\:{\upbeta\:},\:{\updelta\:}\:$are regularization parameters that penalize model complexity.

## Results and discussion

### Mechanical testing

#### Tensile strength and young’s modulus

**Fig. 3:**
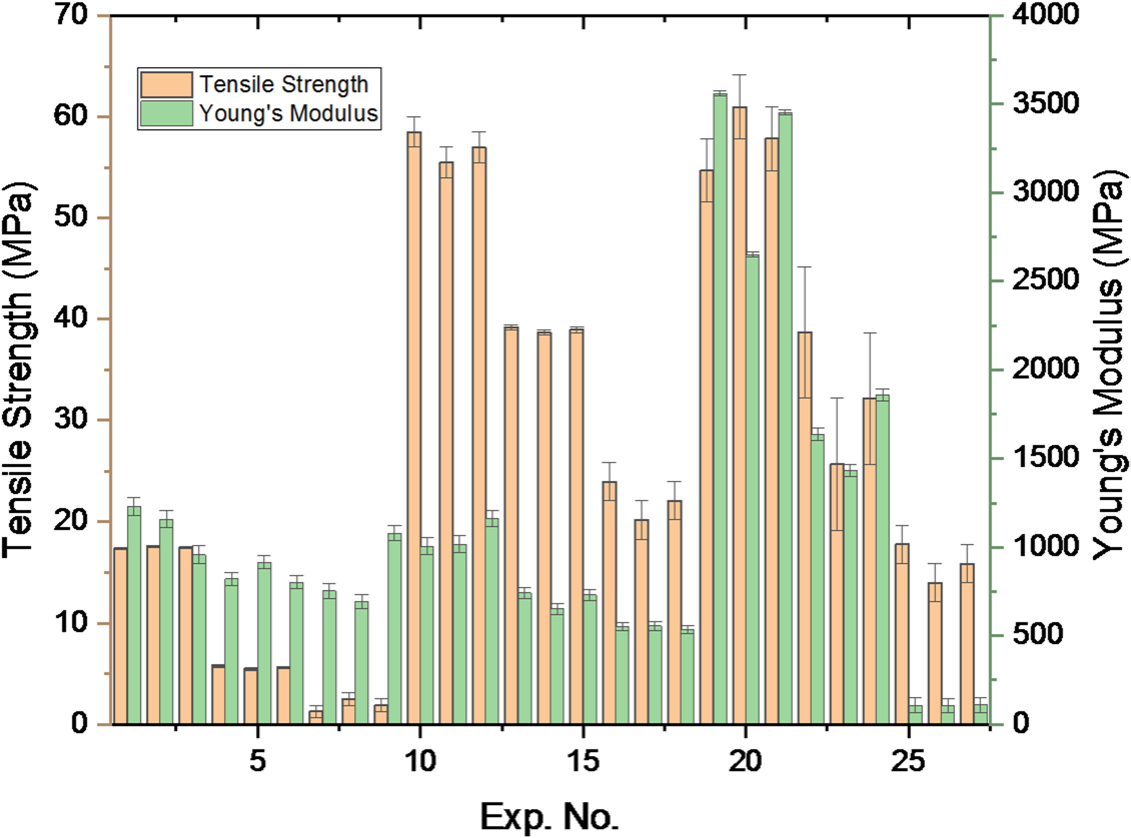
Tensile strength and Young’s modulus vs. experiment number.

The tensile strength and Young’s modulus of PLA/CCB composites were evaluated using an L_27_ orthogonal array designed through the Design of Experiments (DOE) approach, as shown in Table 2. Figure 3 presents the variations across experiments 1 to 27. The minimum tensile strength was observed at Experiments 7–9 with 1.27–2.5 MPa for pure PLA. Similarly, experiments 19–21 exhibit a maximum tensile strength of 54.6-60.99 MPa for 10 wt% PLA/CCB composites. A similar trend was observed for Young’s modulus; experiments 25–27 and 19–21 exhibit a minimal of 105–110 MPa and a maximum of 2652–3561, respectively. These findings demonstrate that both tensile strength and Young’s modulus of PLA/CCB composites are significantly enhanced by increasing the CCB filler content and optimizing the processing conditions, particularly temperature, injection speed, and pressure.

**Fig. 4:**
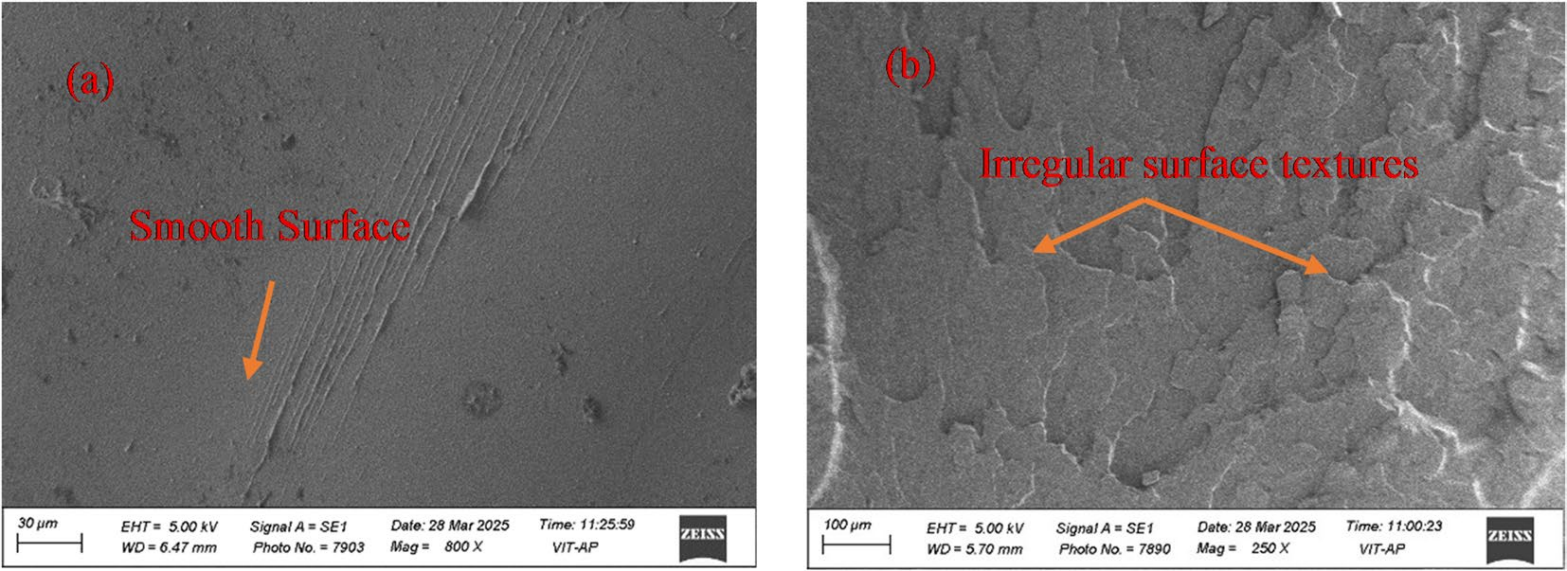
Fractography of (**a**) Pure PLA; (**b**) 10 wt% PLA/CCB composite.

Figure 4a and b illustrates the fractured surface morphology of pure PLA and PLA/CCB composites after tensile test, revealing differences in their failure behavior. Figure 4a shows the fracture surface of pure PLA, which appears smooth, continuous, and homogeneous, characteristic of a relatively ductile fracture^48^. In contrast, Fig. 4b displays the fractured surface of the PLA composite reinforced with 10 wt% CCB, exhibiting a rough, irregular texture with visible surface discontinuities. Incorporating rigid biochar particles disrupts the matrix continuity, creating localized stress concentrations that promote crack initiation and propagation, which is characterized by brittle fracture behaviour^49^.

#### Hardness testing

**Fig. 5:**
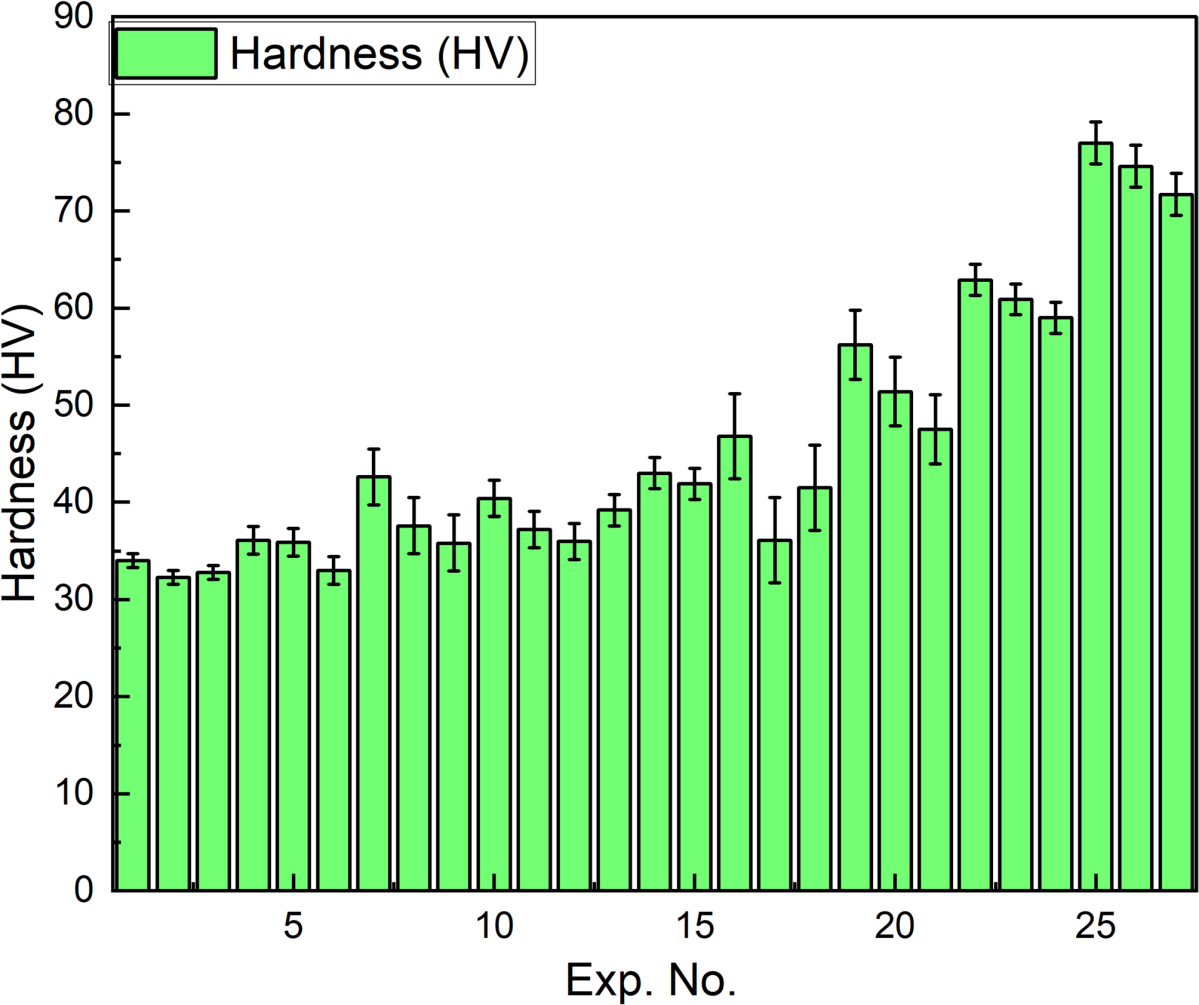
Experimental hardness.

Figure 5 illustrates the hardness of the PLA/CCB composites. Experiments 1–3 exhibit minimal hardness, ranging from 32.3 to 34 HV for pure PLA. However, the experiments 25–27 showed maximum hardness of 71.7 to 77 HV for composites with 10 wt% CCB. These findings clearly demonstrate that increasing the CCB content along with optimizing the processing conditions significantly enhances the hardness of the PLA/CCB composites.

### Statistical analysis

#### Taguchi analysis

The experimentally obtained values of tensile strength, Young’s modulus, and hardness were used to calculate the corresponding signal-to-noise (S/N) ratios for the Taguchi analysis, as presented in Tables 2 and 3. The following sections discuss the optimization of the significant process parameters for each response variable based on the Taguchi method.

##### Tensile strength

Tables 4 and 5 summarize the influence of varying process parameters on tensile strength, considering both the mean values and the larger-is-better signal-to-noise (S/N) ratio. The analysis reveals that composition is the most significant parameter, with the highest delta values of 16.47 and 31.02 for the mean and S/N ratio, respectively. Temperature follows as the second most influential parameter, with delta values of 13.32 and 30.821. In contrast, injection pressure with 3.72 and 6.245 and injection speed with 3.31 and 8.961 exhibit relatively lower delta values for both metrics, indicating a minimal effect on tensile strength. This analysis demonstrated that increasing filler content (composition) and processing temperature have a substantial positive impact on the tensile strength of the composites, while variation in injection pressure and speed has minimal effect.

**Table 4:** Tensile strength response for signal-to-noise ratios.

Level	Composite	Temp	Inj speed	Inj Pressure
1	14.77	31.73	27.16	26.86
2	31.25	25.52	24.66	25.66
3	29.64	18.41	23.85	23.14
Delta	16.47	13.32	3.31	3.72
Rank	1	2	4	3

**Table 5:** Tensile strength response for the mean.

Level	Composite	Temp	Inj speed	Inj Pressure
1	8.327	44.116	23.933	24.117
2	39.350	25.582	26.165	28.514
3	35.316	13.295	32.894	30.362
Delta	31.023	30.821	8.961	6.245
Rank	1	2	3	4

**Fig. 6:**
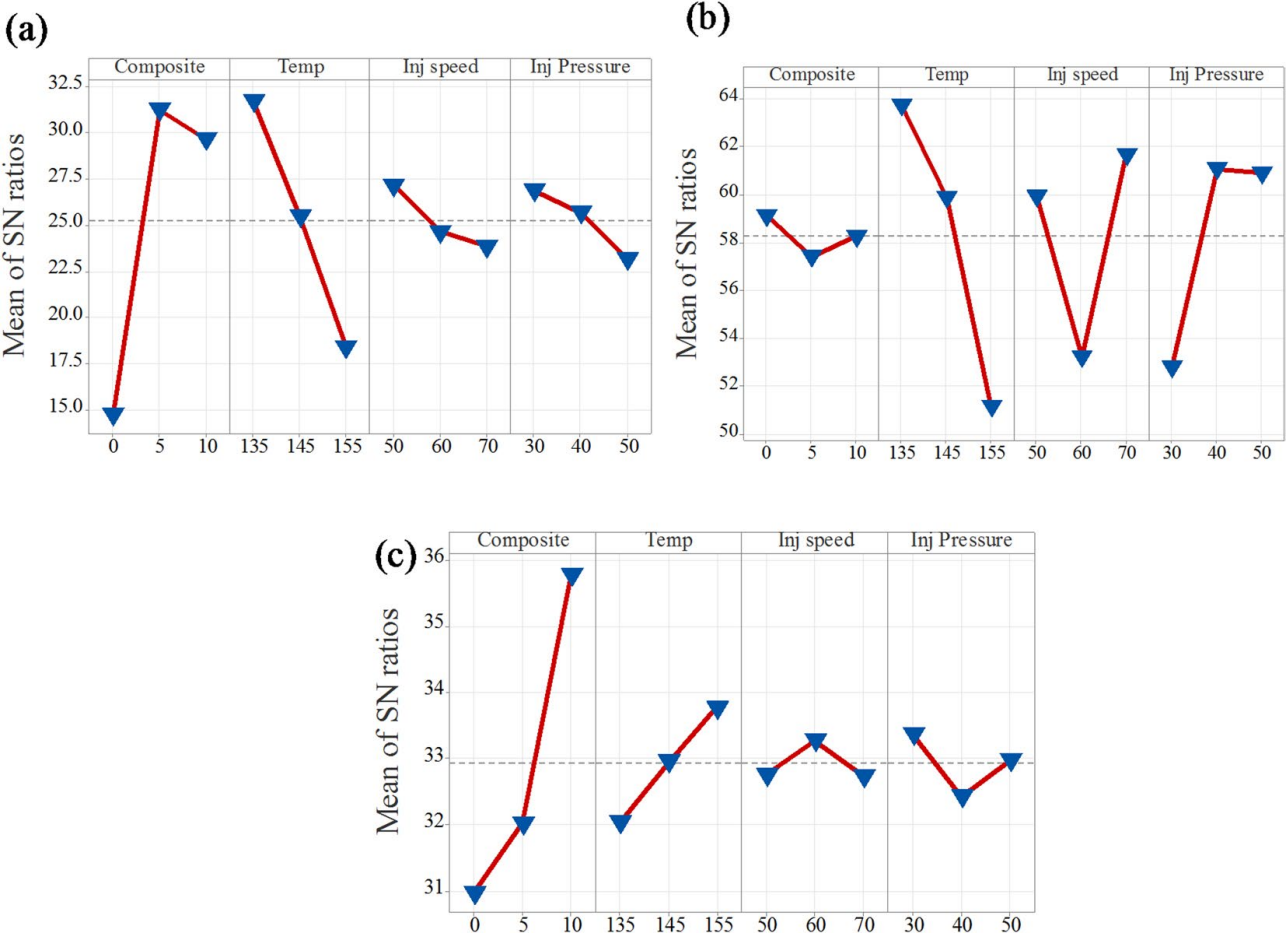
Mean of S/N ratio for (**a**) tensile strength; (**b**) Young’s modulus; (**c**) hardness.

**Fig. 7:**
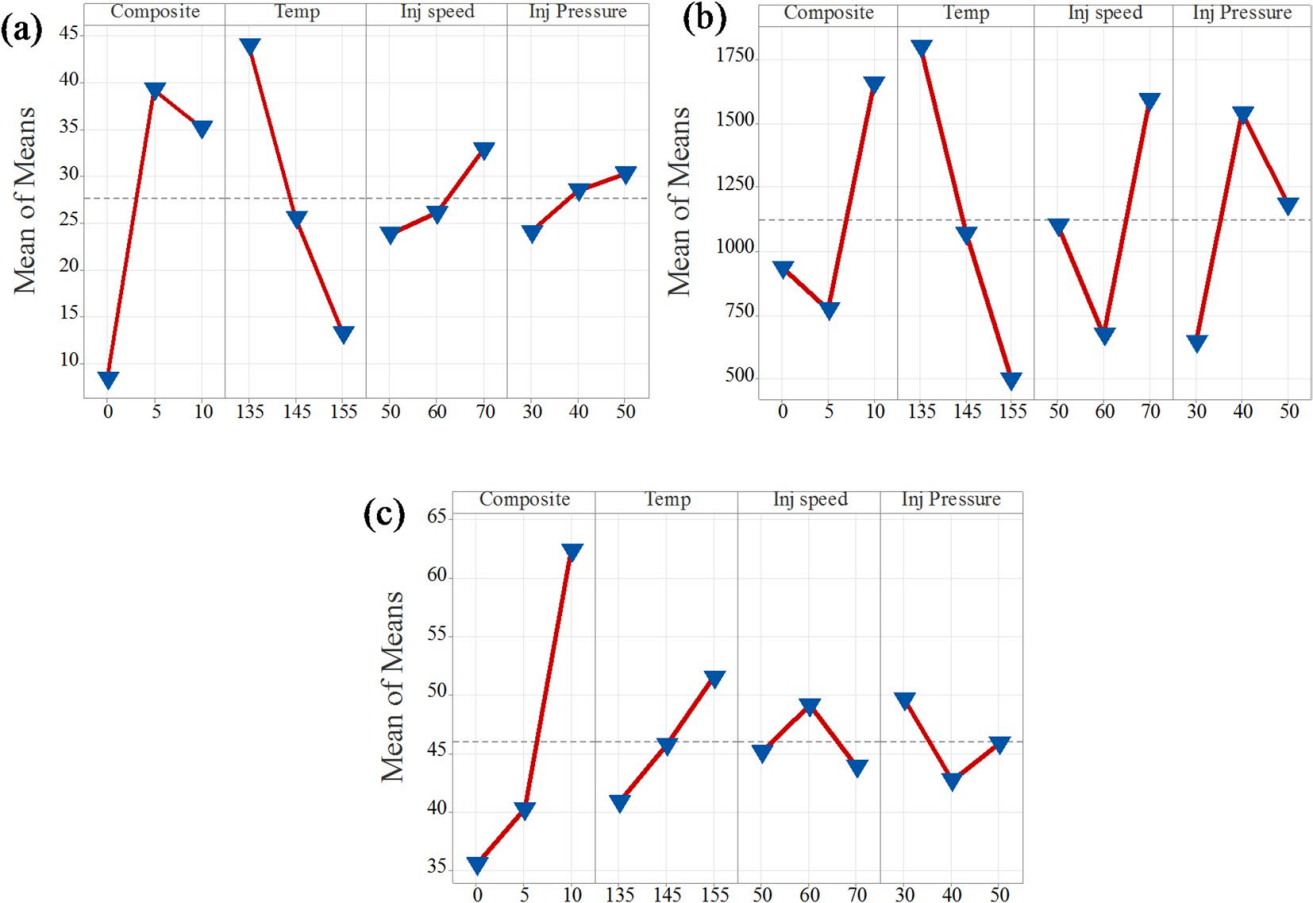
Mean of means for (**a**) tensile strength; (**b**) Young’s modulus; (**c**) hardness.

Figures 6a and 7a present the main effects plots for tensile strength based on the signal-to-noise (S/N) ratio and mean values, respectively. Both plots clearly demonstrate that composition and temperature are the most influential factors affecting tensile strength. From the graphs, it is evident that tensile strength significantly improves as the composition increases from neat PLA to 5% CCB. However, beyond this point, the improvement stabilizes. In the case of temperature, a decline in tensile strength is observed as the processing temperature increases from 135 °C to 155 °C. Additionally, injection pressure and injection speed exhibit moderate effects, displaying nonlinear behavior. In the S/N ratio plot, an increase in injection speed and a reduction in injection pressure lead to a decrease in tensile strength. In contrast, the mean response plot shows an opposite trend, where increasing both injection pressure and injection speed slightly enhances tensile strength. Overall, composition and temperature are confirmed as the most dominant parameters influencing tensile strength, while injection pressure and injection speed have comparatively lesser effects.

##### Young’s modulus

Tables 6 and 7 present the influence of process parameters on Young’s modulus, considering both the mean values and the larger-is-better signal-to-noise (S/N) ratio. The analysis indicates that temperature is the most significant factor affecting Young’s modulus, as reflected by its highest delta values of 12.57 for the S/N ratio and 1300 for the mean. Following this, injection speed, injection pressure, and composition show progressively lower influences, with delta values of 8.43 and 918.9, 8.26 and 894.9, and 1.72 and 885.0, respectively. These results suggest that increasing the processing temperature substantially enhances Young’s modulus, while the effects of injection speed, pressure, and composition are comparatively less pronounced.

**Table 6:** Young’s modulus response table for signal-to-noise ratios.

Level	Composite	Temp	Inj speed	Inj pressure
1	59.12	63.73	59.91	52.81
2	57.40	59.88	53.21	61.07
3	58.25	51.16	61.65	60.90
Delta	1.72	12.57	8.43	8.26
Rank	4	1	2	3

**Table 7:** Young’s modulus response table for mean.

Level	Composite	Temp	Inj speed	Inj Pressure
1	934.8	1800.3	1102.2	644.0
2	772.8	1065.8	672.2	1538.9
3	1657.9	499.4	1591.1	1182.6
Delta	885.0	1300.9	918.9	894.9
Rank	4	1	2	3

The main effects plots for both the signal-to-noise (S/N) ratio and mean values of Young’s modulus are presented in Figs. 6b and 7b. These plots demonstrate that temperature is the most influential factor among all process parameters. Both the S/N ratio and mean response attain their highest values at 135 °C, indicating that this temperature level provides the most robust performance, reinforcing its dominant effect on Young’s modulus. Injection speed emerges as the second most significant factor, with both plots identifying 50 mm/s as the optimal level for achieving better robustness. However, the slightly lower mean response at this setting suggests a trade-off between performance consistency and modulus value. Composite content also contributes notably, where 5% CCB content offers better robustness, while 10% CCB yields the highest mean modulus. Injection pressure, on the other hand, shows the least influence, with minimal variation across different levels, although 40 MPa demonstrates a marginally better performance.

##### Hardness

The hardness of PLA/CCB composites under different processing parameters was evaluated using the signal-to-noise (S/N) ratio and mean values, as summarized in Tables 8 and 9. The analysis indicates that composition is the most influential factor, with the highest delta values of 4.80 for the S/N ratio and 26.79 for the mean ranked as 1. Temperature is the second most significant factor, exhibiting delta values of 1.73 (S/N ratio) and 10.66 (mean). Injection pressure ranks third, with delta values of 0.95 (S/N ratio) and 6.89 (mean), while injection speed shows the least influence, with the lowest delta values of 0.54 (S/N ratio) and 5.19 (mean). Overall, this summary demonstrates that composition has the most dominant effect on enhancing the hardness of PLA/CCB composites, whereas injection speed contributes minimally, highlighting the dominant role of filler content and processing temperature in enhancing the mechanical performance of PLA/CCB composites.

**Table 8:** Hardness response table for Signal-to-Noise ratios.

Level	Composite	Temp	Inj speed	Inj Pressure
1	30.97	32.04	32.76	33.37
2	32.02	32.95	33.27	32.43
3	35.77	33.77	32.73	32.97
Delta	4.80	1.73	0.54	0.95
Rank	1	2	4	3

**Table 9:** Hardness response table for mean.

Level	Composite	Temp	Inj sped	Inj pressure
1	35.57	40.87	45.14	49.61
2	40.23	45.77	49.10	42.72
3	62.36	51.52	43.91	45.82
Delta	26.79	10.66	5.19	6.89
Rank	1	2	4	3

Figures 6c and 7c present the main effects plots for both the signal-to-noise (S/N) ratio and mean values, illustrating the influence of process parameters on the hardness of PLA/CCB composites. These plots show that an increase in composition has the most significant effect on hardness. Temperature and injection pressure show a moderate influence, while injection speed has the least effect. This trend indicates that composition is the dominant factor governing hardness, followed by temperature, injection pressure, and injection speed. The consistent patterns observed in both the S/N ratio and mean response plots further confirm the reliability and validity of this analysis.

#### Analysis of variance (ANOVA)

##### Tensile testing

The ANOVA results presented in Table 10 indicate that the injection molding process parameters statistically affect the tensile strength of PLA/CCB composites, as evidenced by a P-value of 0.000 (*P* < 0.001). This confirms a highly significant influence of the selected process parameters and composition on tensile strength. Among these, composition and temperature were the most impactful factors, contributing 50.43% and 42.67%, respectively. In comparison, injection speed and injection pressure contributed relatively less, at 3.86% and 1.82%, respectively. The remaining 1.22% was attributed to experimental error, suggesting excellent process control and repeatability. Additionally, the regression analysis demonstrated a strong correlation between tensile strength and the process parameters. The model showed a high goodness of fit, with a coefficient of determination (R^2^) of 98.78% and an adjusted R^2^ of 98.23%, indicating the model’s high accuracy, reliability, and suitability for predicting tensile strength outcomes based on the chosen parameters.

**Table 10:** xxx.

Source	DF	Seq SS	Adj SS	Adj MS	F-Value	*P*-Value	Contribution
Composite	2	5121.4	5121.4	2560.71	371.00	0.000	50.43%
Temp	2	4333.2	4333.2	2166.62	313.90	0.000	42.67%
Inj speed	2	391.7	391.7	195.84	28.37	0.000	3.86%
Inj Pressure	2	185.3	185.3	92.63	13.42	0.000	1.82%
Error	18	124.2	124.2	6.90			1.22%
Total	26	10155.9					100.00%

R^2^-sq = 98.78%, R^2^-sq.(adj) = 98.23%.

Regression Eq. 

22 $$\begin{gathered} {\text{TS}}\,=\,{\text{27}}.{\text{664}}\, - \,{\text{19}}.{\text{338}}{{\text{F}}_{\text{1}}}+{\text{11}}.{\text{686}}{{\text{F}}_{\text{2}}}+{\text{7}}.{\text{652}}{{\text{F}}_{\text{3}}}+{\text{16}}.{\text{452}}{{\text{T}}_{\text{1}}} \hfill \\ - {\text{2}}.0{\text{83}}{{\text{T}}_{\text{2}}} - {\text{14}}.{\text{369}}{{\text{T}}_{\text{3}}} - {\text{3}}.{\text{731}}{{\text{S}}_{\text{1}}} - {\text{1}}.{\text{499}}{{\text{S}}_{\text{2}}} \hfill \\ +{\text{5}}.{\text{23}}0{{\text{S}}_{\text{3}}} - {\text{3}}.{\text{548}}{{\text{P}}_{\text{1}}}+0.{\text{85}}0{{\text{P}}_{\text{2}}}+{\text{2}}.{\text{697}}{{\text{P}}_{\text{3}}} \hfill \\ \end{gathered}$$

Here, F_1,_ F_2,_ F_3_: Composite levels (0%, 5%, 10%), T_1,_ T_2,_ T_3_: Temperature levels (135 °C, 145 °C, 155 °C), S_1,_ S_2,_ S_3_: Injection speed levels (50, 60, 70 mm/s), P_1,_ P_2,_ P_3_: Injection pressure levels (30, 40, 50 bar), and TS: Tensile Strength (MPa).

**Fig. 8:**
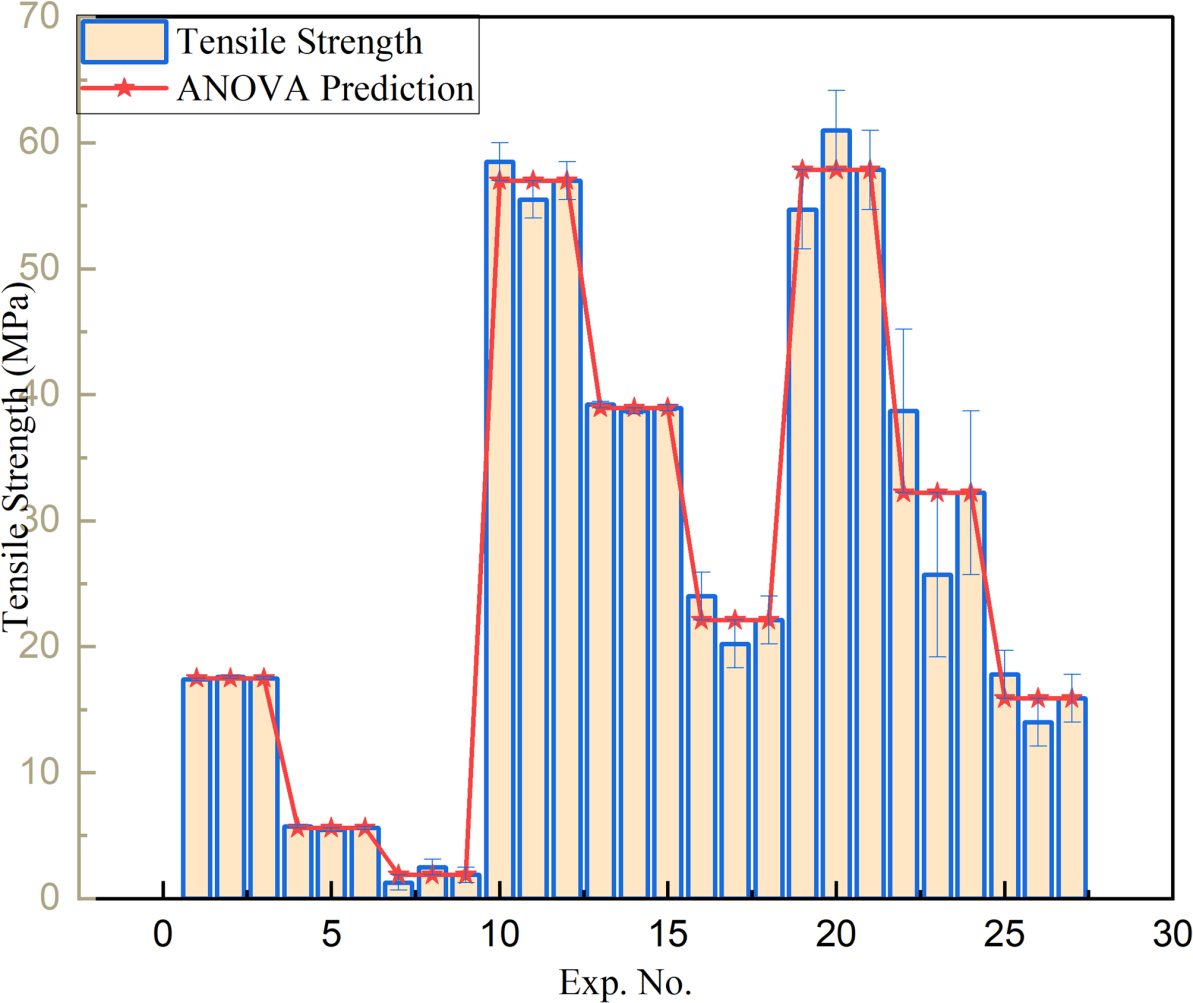
Tensile strength prediction accuracy based on ANOVA model.

Figure 8 presents a comparison between the experimentally measured tensile strength values and those predicted using the ANOVA-based regression equation (Eq. 22). The comparison reveals that most of the experimental values fall within a 10% error margin, indicating strong agreement between the experimental results and the model predictions. For instance, tensile strength values around 17.5 MPa show minimal deviations of approximately ± 0.57%, while values near 5.6 MPa and 22.1 MPa have percentage errors of ± 2.41% and ± 8.6%, respectively. Similarly, measurements close to 38.95 MPa and 57 MPa exhibit even smaller errors of around ± 0.13% and ± 1.5%, confirming the high accuracy of the model.

However, a few data points show larger deviations. Tensile strengths near 15.9 MPa and 32.2 MPa exhibit higher percentage errors of ± 11.95% and ± 20.19%, respectively, slightly exceeding the 10% threshold and suggesting the need for further investigation. The largest deviation is observed for a value near 1.9 MPa, with an error of approximately ± 32.63%. Despite these few outliers, the majority of the data points demonstrate excellent predictive accuracy, underscoring the robustness and reliability of the regression model in capturing the influence of processing parameters on tensile strength.

**Fig. 9:**
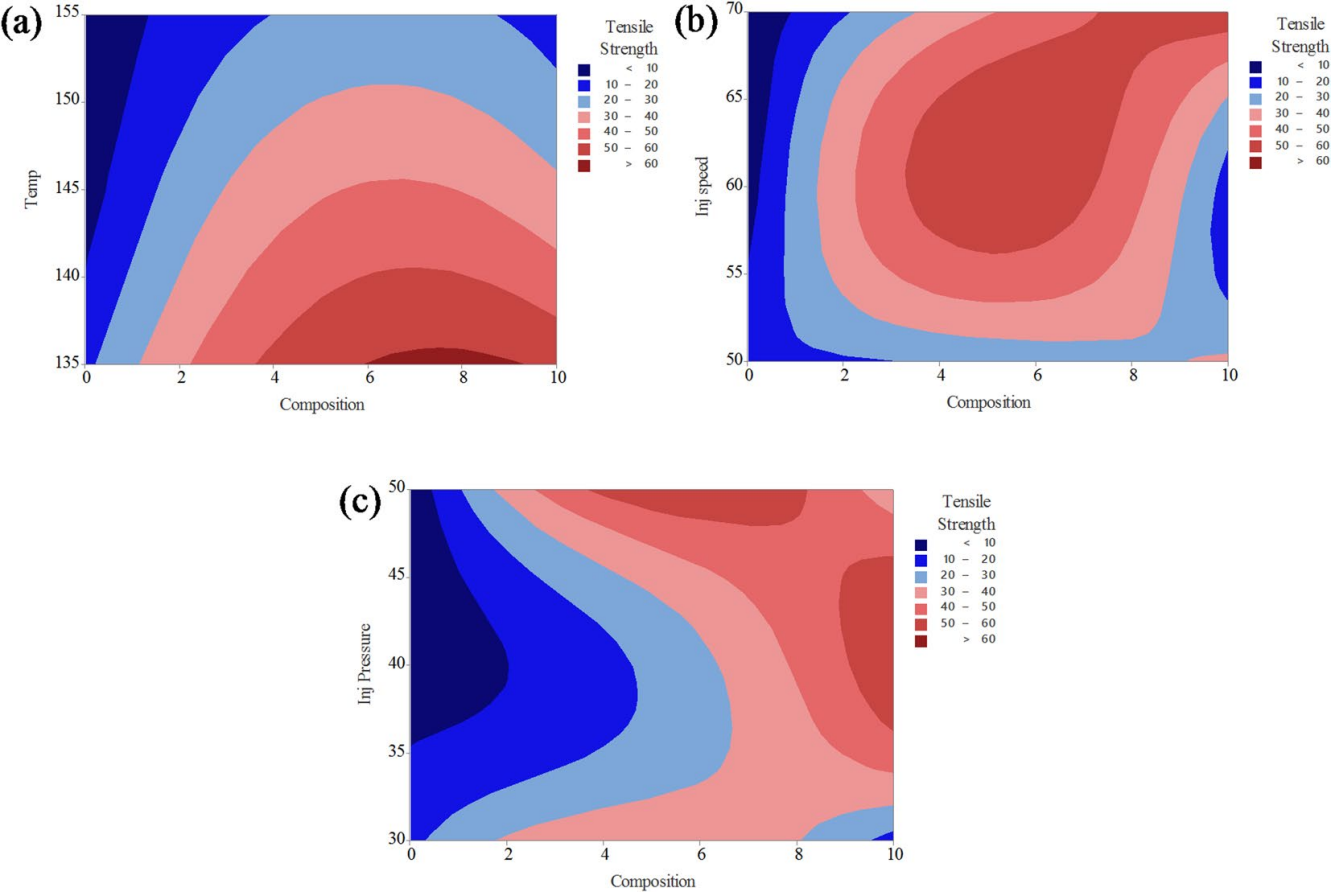
Contour plot analysis for tensile strength on (**a**) composition and temperature; (**b**) composition and injection speed; (**c**) composition and injection pressure.

The contour plots presented in Fig. 9 illustrate the combined effects of two process parameters on the tensile strength of PLA/CCB composites, while keeping the other parameters constant. In Fig. 9a, the interaction between composition and temperature reveals that higher tensile strength values (above 60 MPa, shown in red) are achieved when the composition ranges between 4% and 8% and the processing temperature lies between 135 °C and 145 °C. Conversely, lower tensile strength values (below 30 MPa), represented by blue regions, occur at low filler content combined with higher temperatures. Figure 9b depicts the interaction between composition and injection speed, showing that maximum tensile strength is obtained when the composition is between 5% and 9% and the injection speed ranges from 60 to 65 mm/s. Regions of lower strength (less than 30 MPa) are observed at both the lower and higher ends of composition and injection speed, appearing as dark and light blue zones. In Fig. 9c, the combined effect of composition and injection pressure indicates that tensile strength exceeds 60 MPa (highlighted in red) when the composition is above 6% and the injection pressure is maintained between 35 and 45 bar. Low-strength areas are predominantly located in regions with lower composition and lower pressure values. The color gradients, ranging from dark blue (lowest strength) to red (highest strength), effectively demonstrate how optimizing these process parameters can enhance the mechanical performance of PLA/CCB composites.

**Fig. 10:**
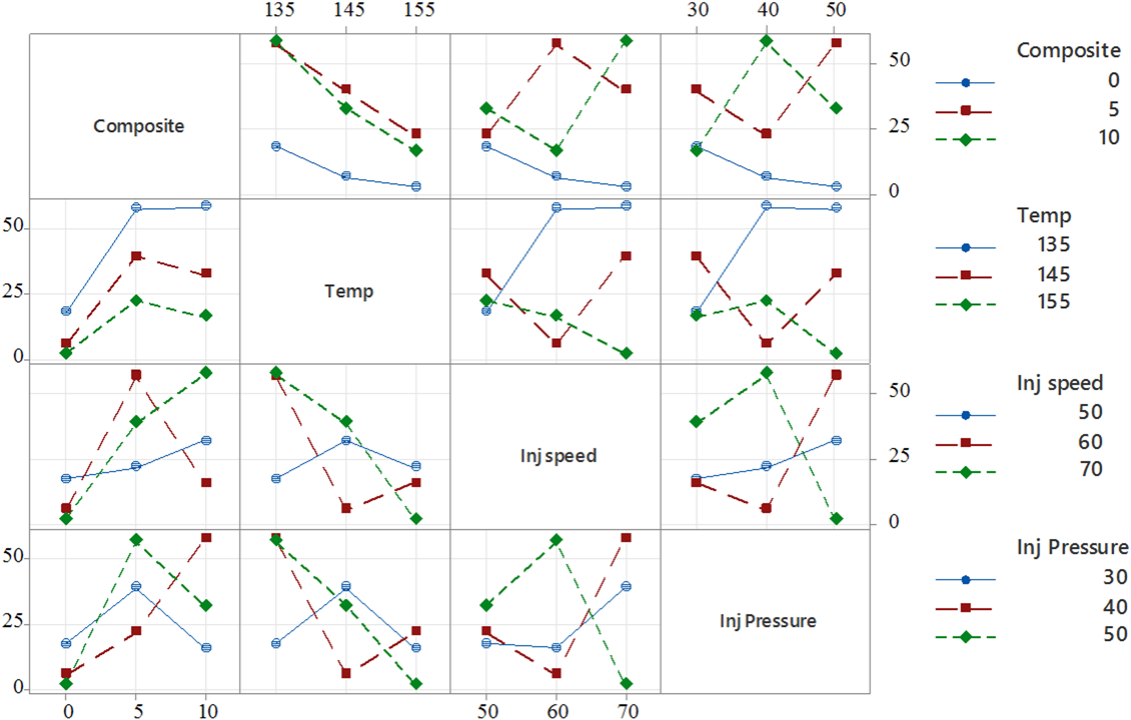
Interaction plot for tensile strength.

Figure 10 presents the interaction plots, illustrating how factors such as composition, temperature, injection speed, and injection pressure interactively influence the tensile strength of PLA/CCB composites. For example, in the plot comparing temperature and composition, the tensile strength increases from approximately 10 to over 50 MPa at 135 °C as the filler content rises from 0% to 10%. However, this trend reverses at higher temperatures; at 155 °C, increasing the composite content causes the tensile strength to drop from around 45 MPa to below 20 MPa, highlighting a significant interaction between these two parameters. Similarly, the subplot of injection speed versus injection pressure shows that tensile strength peaks at an injection speed of 60 mm/s and a pressure of 40 bar, while deviating from this combination results in a sharp decline, indicating the presence of non-linear interactions. The presence of non-parallel lines in most plots further confirms that the effect of one factor is heavily dependent on the level of another. These findings underscore the importance of considering parameter interactions collectively when optimizing processing conditions, rather than evaluating factors in isolation.

##### Young’s modulus

The ANOVA results summarized in Table 11 indicate that the injection molding process parameters have a statistically significant effect on the Young’s modulus of PLA/CCB composites, as evidenced by a P-value of 0.000 (*P* < 0.001). This confirms a highly significant influence of both the process parameters and composition on the stiffness of the composites. Among these factors, temperature and composition were identified as the most influential, contributing 38.58% and 20.14%, respectively. Injection speed and injection pressure also had notable effects, with contributions of 19.17% and 18.41%, respectively. Only 3.72% of the variation was attributed to experimental error, indicating excellent process control and repeatability. In addition, the regression model demonstrated a strong relationship between Young’s modulus and the process parameters. The model showed a high degree of accuracy and reliability, with a coefficient of determination (R^2^) of 97.95% and an adjusted R^2^ of 97.24%, confirming that the selected process parameters provided a good predictive fit for estimating the Young’s modulus of PLA/CCB composites.

**Table 11:** Analysis of variance (ANOVA) for young’s modulus of PLA/CCB composites.

Source	DF	Seq SS	Adj SS	Adj MS	F-Value	*P*-Value	Contribution
Composite	2	3,996,848	3,996,848	1,998,424	48.78	0.000	20.14%
Temp	2	7,657,410	7,657,410	3,828,705	93.45	0.000	38.58%
Inj speed	2	3,804,482	3,804,482	1,902,241	46.43	0.000	19.17%
Inj pressure	2	3,654,019	3,654,019	1,827,010	44.59	0.000	18.41%
Error	18	737,470	737,470	40,971			3.72%
Total	26	19,850,229					100.00%

R-sq = 96.28%, R-sq.(adj) = 94.64%.

Regression Eq. 

23 $$\begin{gathered} {\text{YM}}\,=\,{\text{1121}}.{\text{8}}\, - \,{\text{187}}.0{{\text{F}}_{\text{1}}} - {\text{349}}.0{{\text{F}}_{\text{2}}}\,+\,{\text{536}}.0{{\text{F}}_{\text{3}}}\,+\,{\text{678}}.{\text{4}}{{\text{T}}_{\text{1}}} \hfill \\ - {\text{56}}.0{{\text{T}}_{\text{2}}}\, - \,{\text{622}}.{\text{4}}{{\text{T}}_{\text{3}}}\, - \,{\text{19}}.{\text{6}}{{\text{S}}_{\text{1}}} - {\text{449}}.{\text{6}}{{\text{S}}_{\text{2}}}\, \hfill \\ +\,{\text{469}}.{\text{2}}{{\text{S}}_{\text{3}}} - {\text{477}}.{\text{8}}{{\text{P}}_{\text{1}}}+{\text{417}}.{\text{1}}{{\text{P}}_{\text{2}}}+{\text{6}}0.{\text{7}}{{\text{P}}_{\text{3}}} \hfill \\ \end{gathered}$$

Here, F_1,_ F_2,_ F_3_: Composite levels (0%, 5%, 10%), T_1,_ T_2,_ T_3_: Temperature levels (135 °C, 145 °C, 155 °C), S_1,_ S_2,_ S_3_: Injection speed levels (50, 60, 70 mm/s), P_1,_ P_2,_ P_3_: Injection pressure levels (30, 40, 50 bar), and YM: Young’s modulus (MPa).

**Fig. 11:**
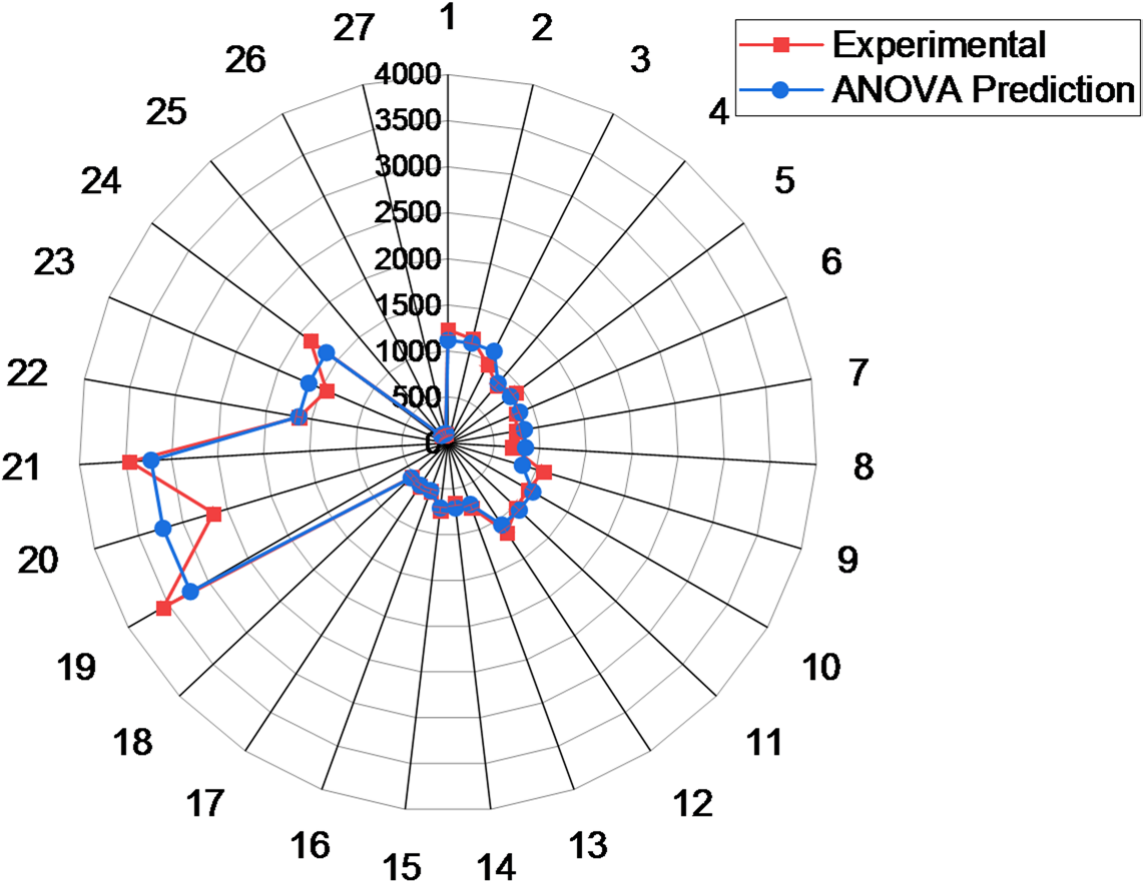
Young’s modulus prediction accuracy based on ANOVA model.

Figure 11 compares the experimentally obtained Young’s modulus values with those predicted by the ANOVA-based regression Eq. (23). The percentage errors between the predicted and actual values were calculated to assess the model’s accuracy. Overall, most deviations fell within an acceptable range, confirming the strong predictive capability of the regression model. For instance, predicted values around 1115.79 MPa exhibited percentage errors between ± 3.88% and ± 14.09%, while predictions near 846.26 MPa showed errors ranging from ± 3.04% to ± 8.14%. Similarly, for values around 1062.41 MPa and 708.21 MPa, the deviations remained within ± 10%. Even at higher modulus values such as 3222.63 MPa, the model maintained reasonable accuracy, with errors ranging from ± 7.18% to ± 17.71%. Notably, for the lowest predicted modulus value of 107.97 MPa, the percentage error was as low as ± 0.03%. These findings confirm the reliability and robustness of the ANOVA-based regression model in accurately estimating Young’s modulus across a broad range of processing conditions for PLA/CCB composites.

**Fig. 12:**
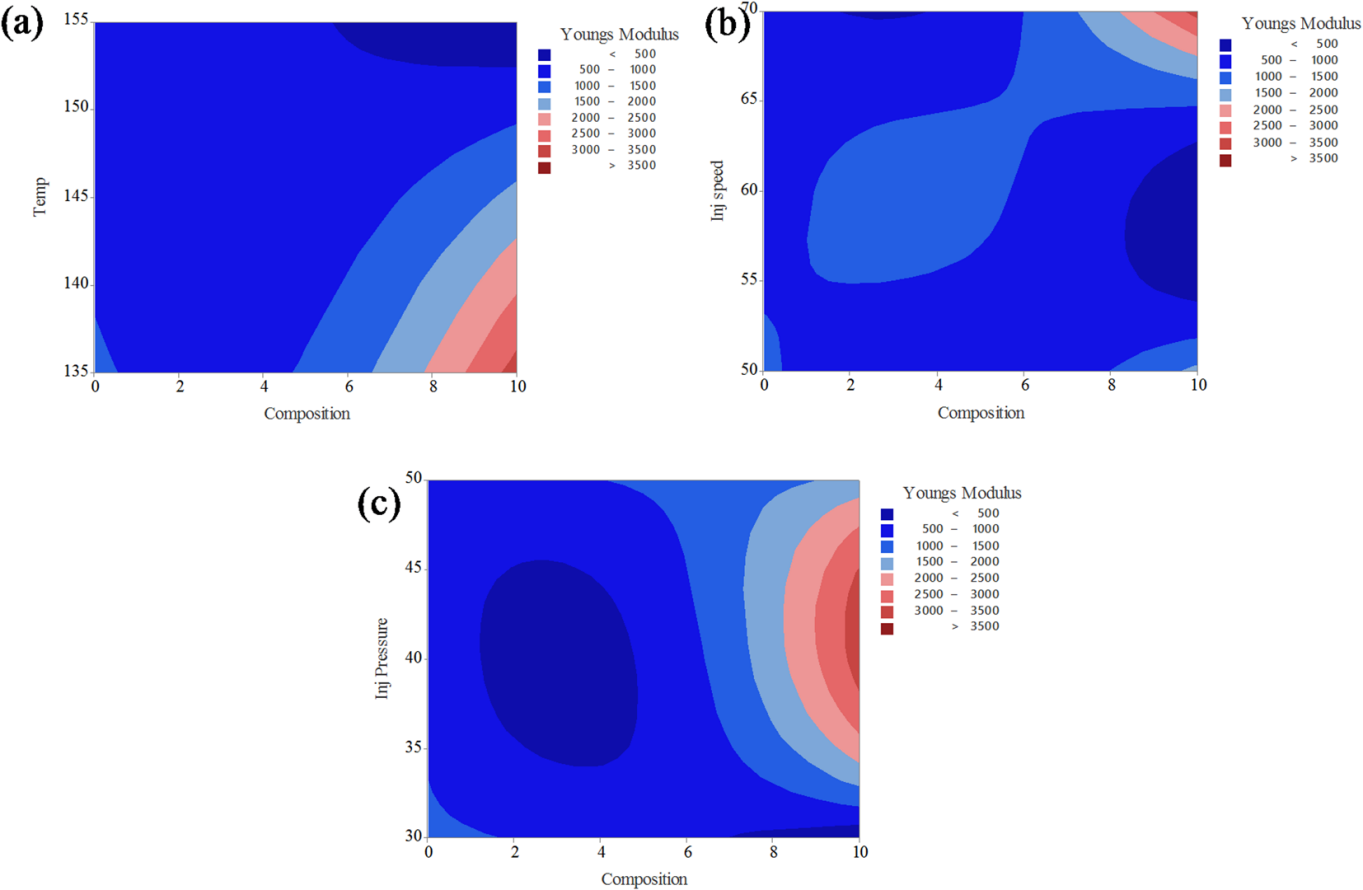
Contour plot analysis for Young’s modulus on (**a**) composition and temperature; (**b**) composition and injection speed; (**c**) composition and injection pressure.

The contour plots in Fig. 12 illustrate the combined effects of two process parameters on the Young’s modulus of PLA composites reinforced with CCB, while keeping the other parameters constant. In Fig. 12a, the interaction between composition and temperature shows that Young’s modulus significantly improves at higher filler contents, particularly above 7%, and at lower processing temperatures between 135 °C and 140 °C, where modulus values exceed 3000 MPa (represented by the red zone). In contrast, most of the plot remains dark blue, indicating modulus values below 1500 MPa, especially at lower compositions and higher temperatures. Figure 12b displays the combined effect of composition and injection speed, where moderately high modulus values (ranging from 2000 to 3000 MPa, shown in light red to red shades) are observed at higher compositions (8–10%) and injection speeds around 65 mm/s. However, a large portion of the plot remains blue, representing modulus values below 2000 MPa. In Fig. 12c, the interaction between composition and injection pressure reveals that Young’s modulus exceeds 3000 MPa when the composition is above 8% and the injection pressure is maintained between 40 and 45 bar. Similar to the other plots, the majority of this contour remains in the lower blue regions, indicating values under 2000 MPa. The color gradient, ranging from dark blue (< 500 MPa) to dark red (> 3500 MPa), effectively highlights the positive influence of increased CCB content and optimized processing parameters on enhancing the stiffness of PLA/CCB composites.

**Fig. 13:**
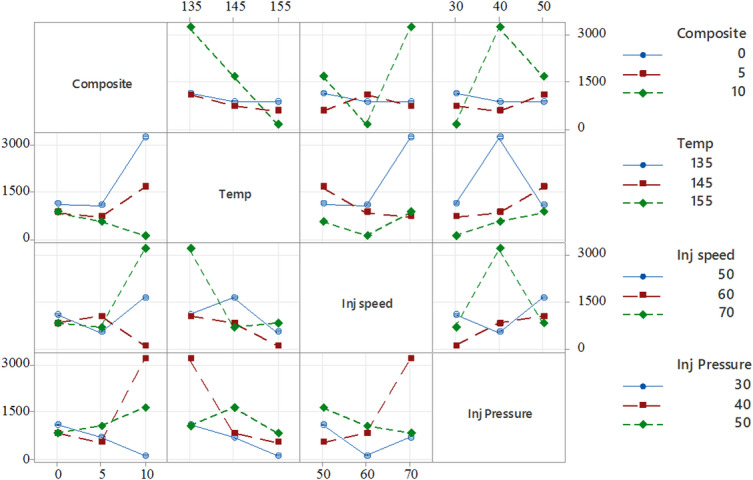
Interaction plot for Young’s modulus.

Figure 13 presents the interaction plots, demonstrating how factors such as composition, temperature, injection speed, and injection pressure collectively influence the Young’s modulus of PLA/CCB composites. For instance, in the plot comparing temperature and composition, the Young’s modulus increases sharply from approximately 1200 MPa to over 3000 MPa at 135 °C as the filler content rises from 0% to 10%. However, this trend reverses at 155 °C, where increasing the composite content results in a sharp decline in modulus, dropping from around 1200 MPa to nearly zero, which clearly indicating a strong interaction between these two parameters. Similarly, in the injection speed versus injection pressure subplot, the Young’s modulus reaches its peak at an injection speed of 60 mm/s and a pressure of 40 bar but decreases considerably at other parameter combinations, highlighting the presence of non-linear interactions. The appearance of non-parallel and intersecting lines across multiple plots further confirms that the effect of one process factor is highly dependent on the levels of the others. These findings emphasize the necessity of considering the combined interactions among processing parameters when optimizing for maximum stiffness, rather than evaluating each factor in isolation.

##### Hardness

The ANOVA results presented in Table 12 indicate that the injection molding process parameters have a statistically significant effect on the hardness of PLA/CCB composites, as confirmed by a P-value of 0.000 (*P* < 0.001). This highlights a strong influence of both the selected processing parameters and composition on the hardness performance of the composites. Among these factors, composition emerged as the most dominant, contributing 78.23% to the total variation, followed by temperature with a contribution of 10.87%. Injection speed and injection pressure also had measurable, though comparatively smaller, effects, contributing 2.81% and 4.55%, respectively. The remaining 3.55% was attributed to experimental error, indicating good process stability and repeatability. In addition, the regression analysis demonstrated a strong correlation between hardness and the processing parameters. The regression model, represented by Eq. (24), confirmed its robustness, achieving a coefficient of determination (R^2^) of 96.45% and an adjusted R^2^ of 94.87%. These values indicate high predictive accuracy and reliability in estimating the hardness of PLA/CCB composites under various processing conditions.

**Table 12:** Analysis of variance (ANOVA) for hardness of PLA/CCB composites.

Source	DF	Seq SS	Adj SS	Adj MS	F-value	*P*-value	Contribution
Composite	2	3686.4	3686.4	1843.22	198.33	0.000	78.23%
Temp	2	512.0	512.0	256.02	27.55	0.000	10.87%
Inj speed	2	132.3	132.3	66.14	7.12	0.005	2.81%
Inj Pressure	2	214.3	214.3	107.13	11.53	0.001	4.55%
Error	18	167.3	167.3	9.29			3.55%
Total	26	4712.3					100.00%

R-sq = 96.45%, R-sq.(adj) = 94.87%.

Regression Equation 

24 $$\begin{gathered} {\text{Hardness }}\left( {\text{H}} \right)={\text{46}}.0{\text{52}}\, - \,{\text{1}}0.{\text{485}}{{\text{F}}_{\text{1}}}\, - \,{\text{5}}.{\text{819}}{{\text{F}}_{\text{2}}}\,+\,{\text{16}}.{\text{3}}0{\text{4}}{{\text{F}}_{\text{3}}}\, - \,{\text{5}}.{\text{185}}{{\text{T}}_{\text{1}}}\, - \,0.{\text{285}}{{\text{T}}_{\text{2}}}\, \hfill \\ +\,{\text{5}}.{\text{47}}0{{\text{T}}_{\text{3}}}\, - \,0.{\text{9}}0{\text{7}}{{\text{S}}_{\text{1}}}\,+\,{\text{3}}.0{\text{48}}{{\text{S}}_{\text{2}}}\, - \,{\text{2}}.{\text{141}}{{\text{S}}_{\text{3}}}\,+\,{\text{3}}.{\text{559}}{{\text{P}}_{\text{1}}}\, - \,{\text{3}}.{\text{33}}0{{\text{P}}_{\text{2}}}\, - \,0.{\text{23}}0{{\text{P}}_{\text{3}}}. \hfill \\ \end{gathered}$$

Here, F_1,_ F_2,_ F_3_: Composite levels (0%, 5%, 10%), T_1,_ T_2,_ T_3_: Temperature levels (135 °C, 145 °C, 155 °C), S_1,_ S_2,_ S_3_: Injection speed levels (50, 60, 70 mm/s), P_1,_ P_2,_ P_3_: Injection pressure levels (30, 40, 50 bar), and H: Hardness (HV).

**Fig. 14:**
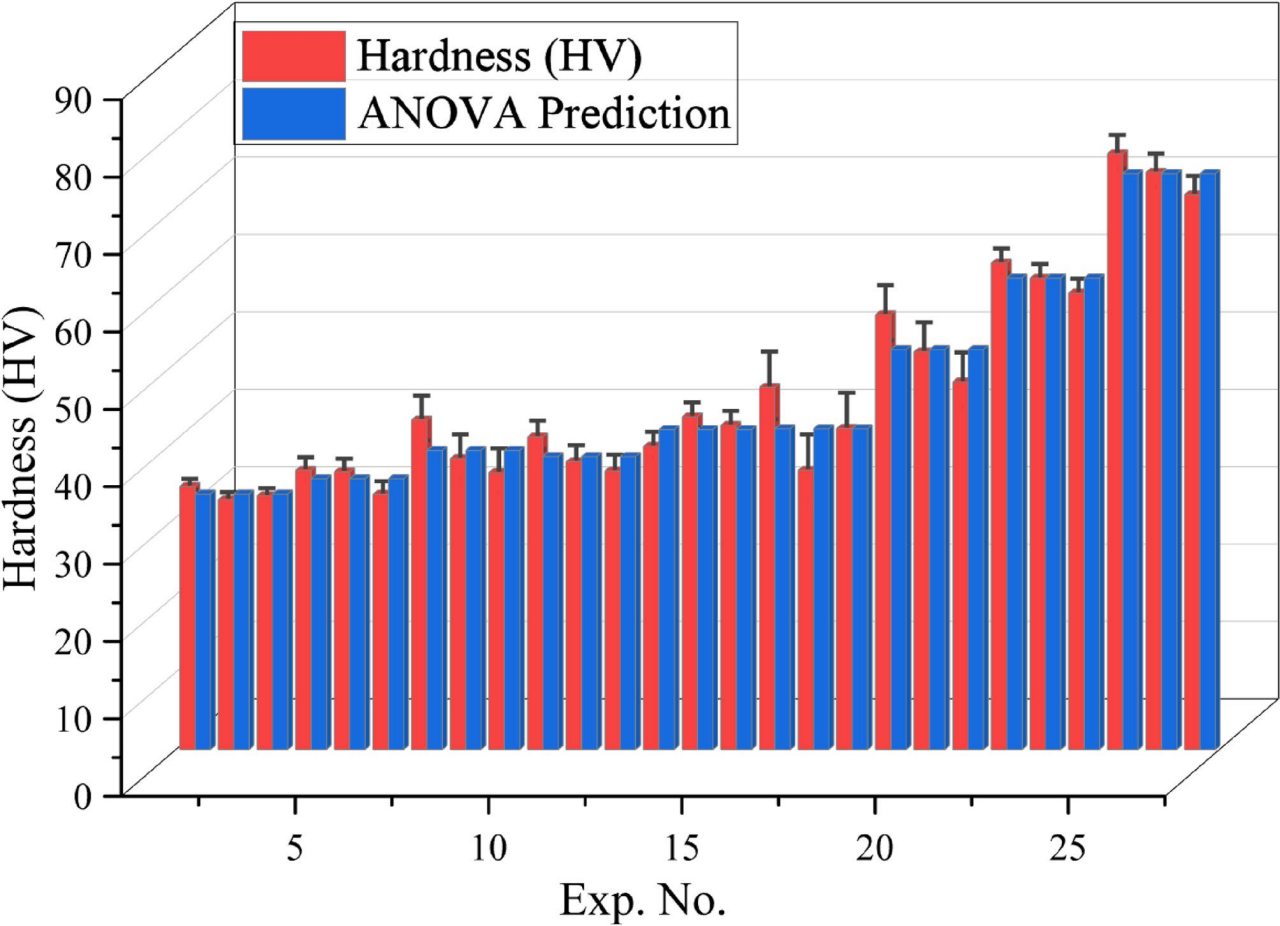
Hardness prediction accuracy based on ANOVA model.

Figure 14 presents a comparison between the experimentally measured and ANOVA-predicted hardness values for the PLA/CCB composites. The experimental hardness values ranged from 32.3 to 77, while the predicted values varied between 33.03 and 74.43. The percentage deviations between experimental and predicted results were generally below 10%, indicating strong agreement and high predictive accuracy of the regression model. For instance, an experimental hardness of 34 exhibited a deviation of approximately 2.96% from the predicted value of 33.03, while hardness values around 35.9 showed deviations close to 2.57%. Although a few data points displayed slightly higher deviations—such as an experimental value of 46.8 compared to a predicted 41.47—the majority of values remained well within acceptable limits. These findings confirm that the ANOVA-based regression model effectively captures the influence of processing parameters on the hardness of PLA/CCB composites and provides reliable predictions. This demonstrates the model’s practical value for process optimization, property prediction, and quality control in composite manufacturing.

**Fig. 15:**
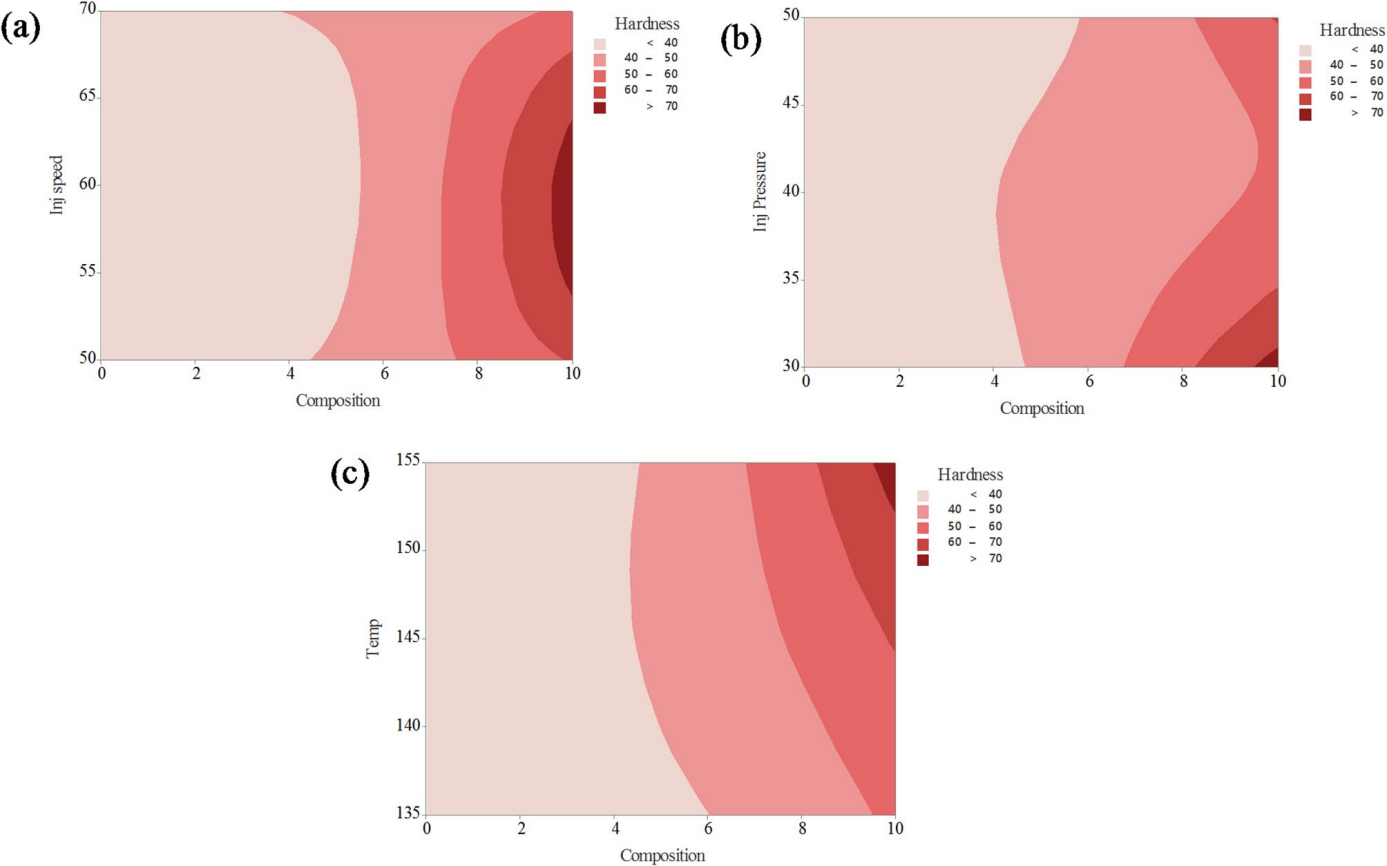
Contour plot analysis for hardness on (**a**) composition and injection speed; (**b**) Composition and injection pressure; (**c**) composition and temperature.

The contour plots in Fig. 15a–c illustrate the combined effects of two process parameters on the hardness of PLA composites reinforced with CCB, while keeping the other parameters constant. In Fig. 15a, the interaction between composition and injection speed reveals that hardness steadily increases as the CCB content exceeds 6%, reaching maximum values above 70 HV (indicated by dark red regions) at around 9–10% composition, largely independent of injection speed. Most of the lower composition region remains in light pink shades, representing hardness values below 50 HV. Figure 15b shows the combined effect of composition and injection pressure, where hardness values greater than 70 HV are achieved when the composition exceeds 8% and the injection pressure is maintained between 40 and 50 bar. In contrast, areas with composition below 6% consistently record hardness values under 50 HV. Similarly, Fig. 15c illustrates the interaction between composition and temperature, indicating that hardness exceeds 70 HV when the CCB content is above 8% and the processing temperature ranges between 140 °C and 150 °C. The color gradient, shifting from light pink (< 40 HV) to dark red (> 70 HV), consistently demonstrates that increasing CCB content has a pronounced positive effect on hardness across all processing conditions. Additionally, optimized ranges of injection speed, pressure, and temperature further enhance the material’s hardness performance, reinforcing the role of composition as the most dominant influencing factor.

**Fig. 16:**
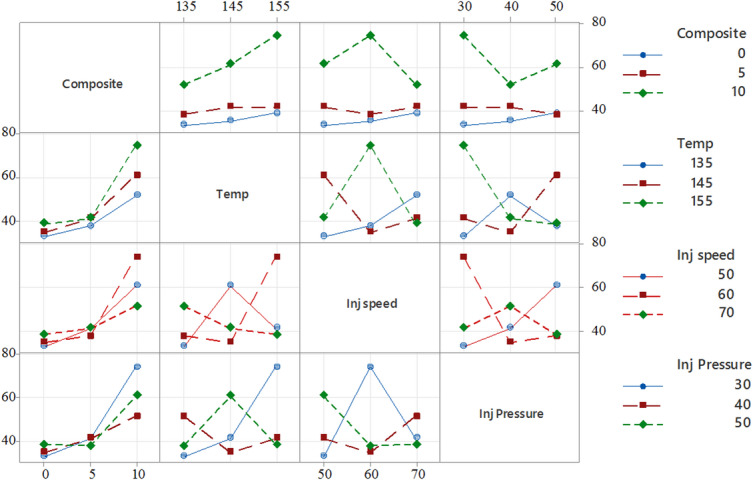
Interaction plot for hardness.

Figure 16 presents the interaction plots, illustrating how composition, temperature, injection speed, and injection pressure collectively influence the hardness of PLA/CCB composites. In the plot comparing temperature and composition, hardness increases consistently with both parameters. At 155 °C, raising the composite content from 0% to 10% results in a sharp rise in hardness from approximately 40 HV to nearly 80 HV, indicating a strong synergistic interaction between these two factors. In contrast, the plot for injection speed versus injection pressure displays a more complex relationship. Peak hardness is achieved at an injection speed of 60 mm/s and an injection pressure of 40 MPa, while other combinations produce more varied and less predictable responses. The presence of intersecting and non-parallel lines across the interaction plots confirms that the effect of one process parameter is highly dependent on the levels of the others. These trends emphasize that maximizing the hardness of PLA/CCB composites requires a simultaneous and balanced optimization of all processing parameters, rather than adjusting them in isolation.

### Machine learning

#### Tensile strength

**Fig. 17:**
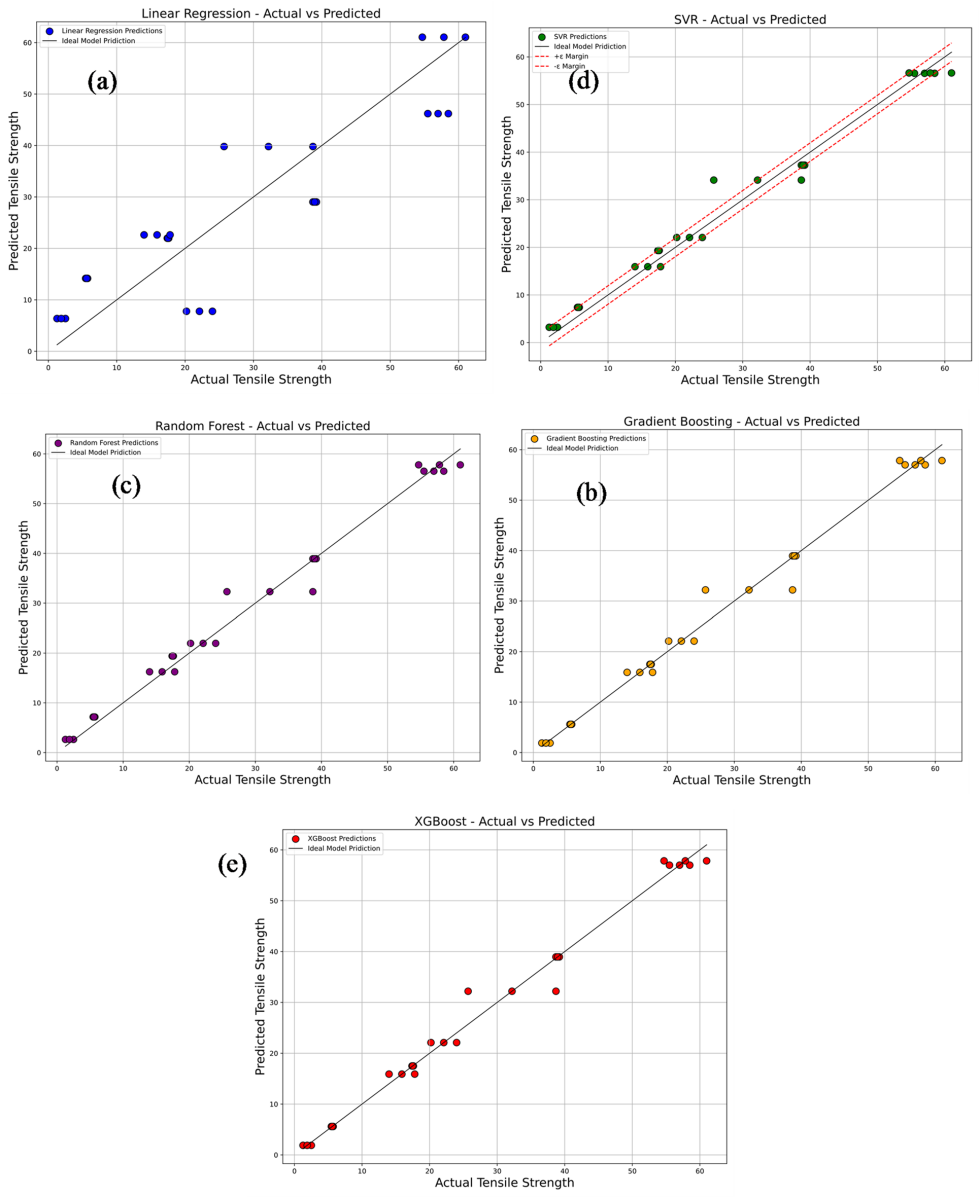
Comparison of actual vs. predicted tensile strength using machine learning models (**a**) linear regression; (**b**) support vector regression (SVR); (**c**) random forest; (**d**) gradient boosting; (**e**) XG-boost.

Figure 17a–e compares the experimentally measured tensile strength values of PLA/CCB composites with predictions from various machine learning models, including Linear Regression, Support Vector Regression (SVR), Random Forest Regression, Gradient Boosting, and Extreme Gradient Boosting (XG-Boost). In each plot, colored dots represent the model’s predicted values, while a 45° reference line indicates perfect prediction accuracy. In Fig. 17a, the Linear Regression model, represented by blue dots, shows considerable deviations from the ideal line, reflecting its limited ability to capture the non-linear relationships within the data. This is further supported by its low R^2^ value of 0.7965 and high error metrics: MSE of 76.53, RMSE of 8.75, MAE of 7.79, and MAPE of 69.87%. Figure 17b depicts the performance of the SVR model, shown with green dots. Here, predictions align more closely with the diagonal line, although some dispersion remains at the data extremes. The model delivers a significantly improved R^2^ of 0.9829, with reduced errors: MSE of 6.45, RMSE of 2.54, MAE of 1.98, and MAPE of 18.68%. In Fig. 17c, the Random Forest model, represented by purple dots, displays even tighter clustering along the reference line, indicating further enhancement in prediction accuracy. It achieves an R^2^ of 0.9857, MSE of 5.38, RMSE of 2.32, MAE of 1.64, and MAPE of 13.92%. Figure 17d presents the Gradient Boosting model, shown with orange dots, where predictions are closely aligned with the ideal line, reflecting excellent accuracy and generalization. This model records an R^2^ of 0.9878, MSE of 4.60, RMSE of 2.15, MAE of 1.19, and MAPE of 6.67%. Figure 17e illustrates the XG-Boost model’s performance, with red dots almost perfectly aligned along the 45° line. It matches the performance of Gradient Boosting with an R^2^ of 0.9878, MSE of 4.60, RMSE of 2.15, MAE of 1.19, and MAPE of 6.67%, confirming its exceptional predictive capability. Overall, these results highlight that ensemble-based models, particularly Gradient Boosting and XG-Boost deliver superior prediction accuracy and reliability for estimating tensile strength, outperforming simpler models like Linear Regression and SVR.

**Fig. 18:**
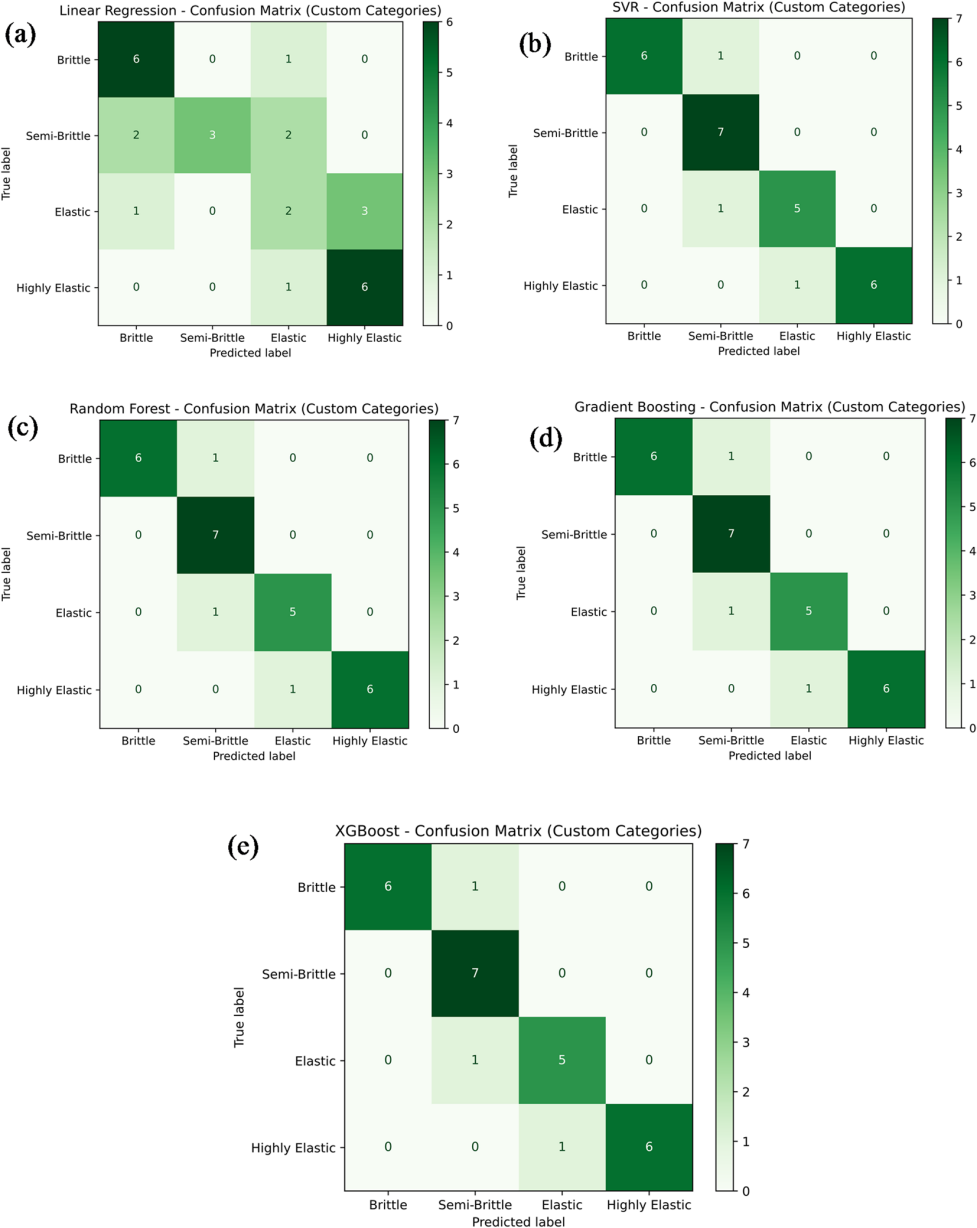
Confusion matrix for machine learning model predicting tensile strength by (**a**) linear regression; (**b**) support vector regression (SVR); (**c**) random forest; (**d**) gradient boosting; (**e**) XG-Boost.

Figure 18a–e presents the classification performance of various machine learning models — namely Linear Regression, Support Vector Regression (SVR), Random Forest Regression, Gradient Boosting, and XG-Boost — in predicting the tensile strength category of PLA/CCB composites based on their material behavior: brittle, semi-brittle, elastic, and highly elastic. In Fig. 18a, the Linear Regression model achieves a classification accuracy of 60.7%, correctly identifying 6 brittle, 3 semi-brittle, 2 elastic, and 6 highly elastic samples. However, several misclassifications are observed, including 1 brittle sample incorrectly classified as elastic, 2 semi-brittle samples misclassified as brittle and elastic, 1 elastic sample classified as brittle, 3 elastic samples as highly elastic, and 1 elastic sample again as highly elastic. In comparison, Fig. 18b–e display the classification results of SVR, Random Forest, Gradient Boosting, and XG-Boost models. Each of these models achieved a notably higher classification accuracy of 85.7%, correctly categorizing 6 brittle, 7 semi-brittle, 5 elastic, and 6 highly elastic samples. The few misclassifications observed included 1 semi-brittle sample classified as brittle, another semi-brittle as elastic, and 1 elastic sample classified as highly elastic. Overall, these figures collectively provide a clear comparison of the classification accuracy and predictive reliability of the tested models. Ensemble learning techniques like Random Forest, Gradient Boosting, and XG-Boost outperformed Linear Regression and SVR, consistently delivering higher classification accuracy across different material behavior categories.

#### Young’s modulus

**Fig. 19:**
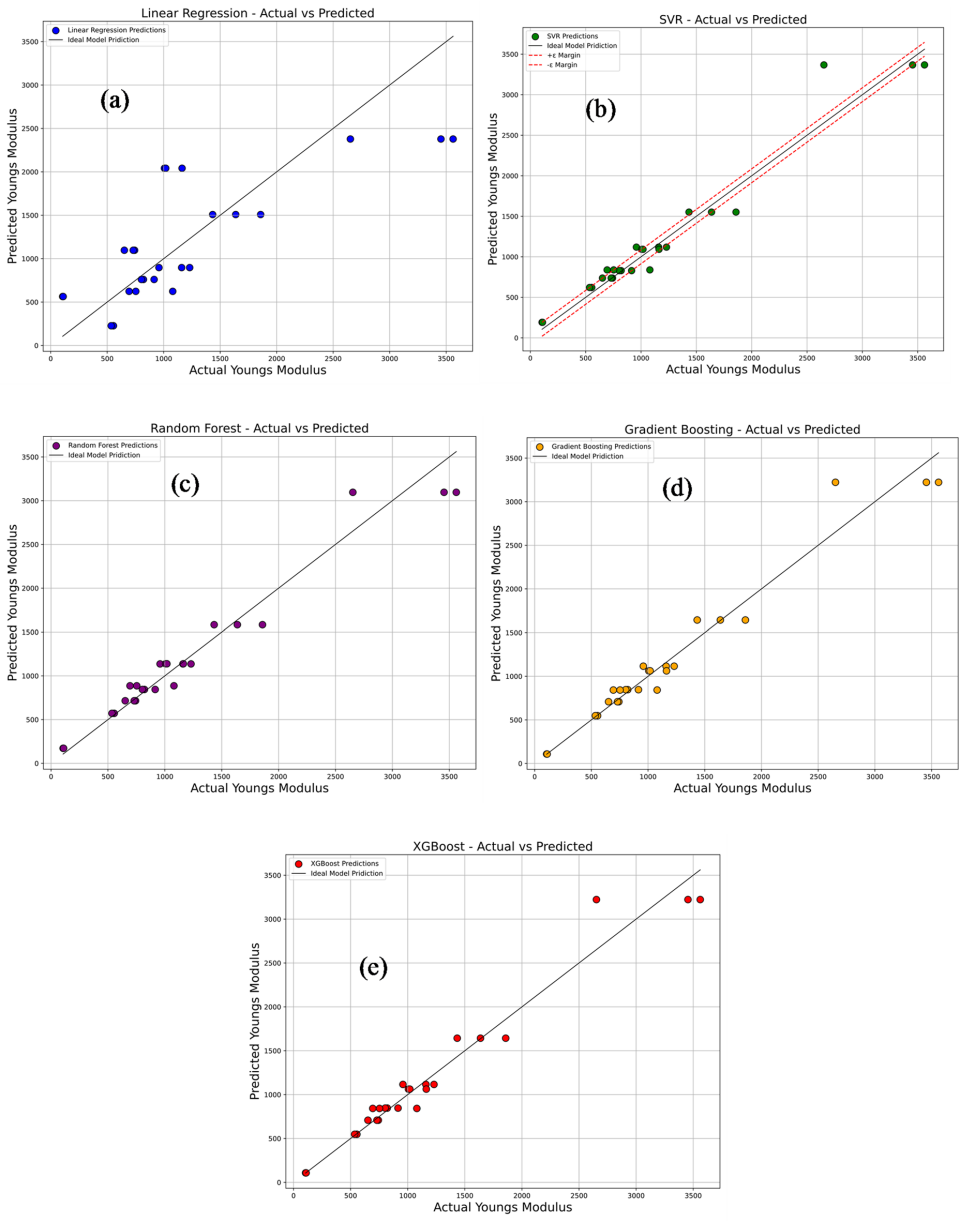
Comparison of actual vs. predicted young’s modulus using machine learning models: (**a**) linear regression; (**b**) support vector regression (SVR); (**c**) random forest; (**d**) gradient boosting; (**e**) XG-Boost.

Figure 19a–e compares the experimentally measured values of Young’s modulus with the predictions obtained from various machine learning models, namely Linear Regression, Support Vector Regression (SVR), Random Forest Regression, Gradient Boosting, and Extreme Gradient Boosting (XG-Boost). In each plot, the predicted values are represented by colored dots plotted against a 45° reference line, which signifies perfect prediction accuracy. As shown in Fig. 19a, the Linear Regression model (blue dots) exhibits noticeable deviations from the reference line, indicating its inability to effectively capture the inherent non-linear relationships present in the dataset. This limitation is evident from its relatively poor performance indicators: an R^2^ of 0.622, MSE of 277,846.6, RMSE of 527.11, MAE of 410.78, and MAPE of 79.60%. In comparison, the SVR model illustrated in Fig. 19b with green dots shows improved alignment with the diagonal, although some dispersion remains. It achieves a notably higher R^2^ value of 0.9562, along with considerably lower error metrics: MSE of 32,194.02, RMSE of 179.43, MAE of 118.87, and MAPE of 17.50%. Further improvement is observed with the Random Forest model in Fig. 19c (purple dots), where the predicted values cluster more tightly around the 45° line. This enhanced performance is reflected by an R^2^ of 0.9579, MSE of 30,921.54, RMSE of 175.85, MAE of 123.73, and MAPE of 15.15%. The Gradient Boosting model, shown in Fig. 19d with orange dots, demonstrates even stronger predictive accuracy, with predictions closely following the ideal line. This model records an R^2^ of 0.9628, MSE of 27,313.71, RMSE of 165.27, MAE of 105.25, and MAPE of 7.72%, indicating excellent generalization and minimal prediction errors. A comparable level of performance is achieved by the XG-Boost model, depicted in Fig. 19e with red dots. Its predicted values almost perfectly overlap the 45° line, and it records identical performance metrics to those of Gradient Boosting. Overall, the results clearly indicate that tree-based ensemble models—particularly Gradient Boosting and XG-Boost—are highly effective in capturing complex non-linear patterns within the data. This is critically important for reliably predicting the mechanical behavior of materials, which can be adversely affected by factors such as thermal degradation and molecular reorientation during the cooling phase. Accurate modeling of these effects is therefore essential to ensure optimal material performance and structural integrity.

**Fig. 20:**
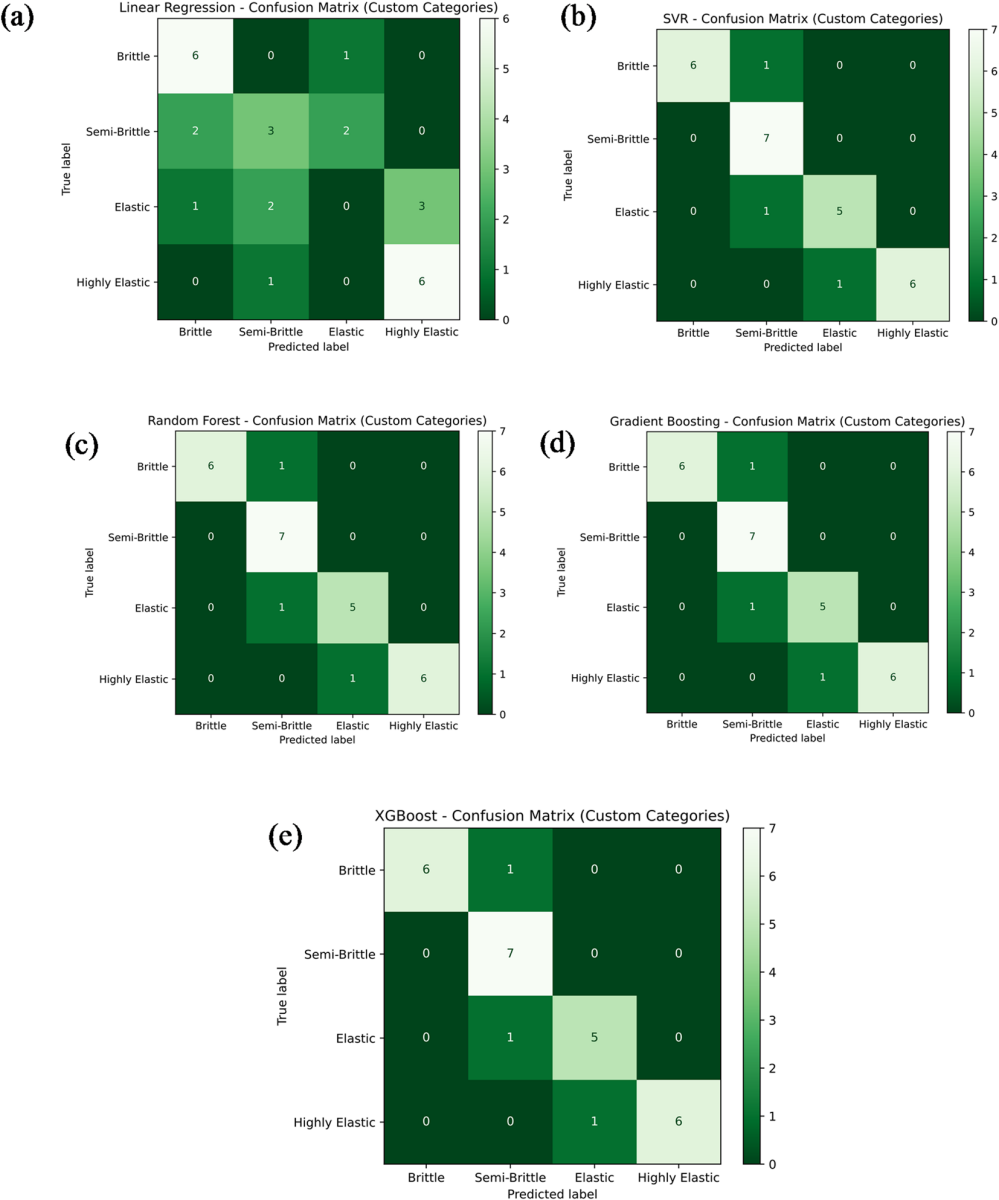
Confusion matrix for the machine learning model predicting Young’s modulus via (**a**) linear regression; (**b**) support vector regression (SVR); (**c**) random forest; (**d**) gradient boosting; e) XG-Boost.

Figure 20a–e presents the classification performance of various machine learning models in predicting Young’s modulus categories for different material types, including brittle, semi-brittle, elastic, and highly elastic. The models evaluated include Linear Regression, Support Vector Regression (SVR), Random Forest Regression, Gradient Boosting, and Extreme Gradient Boosting (XG-Boost). As shown in Fig. 20a, the Linear Regression model correctly classified 6 as brittle, 3 as semi-brittle, and 6 as highly elastic samples, while failing to identify any elastic samples. This resulted in an overall classification accuracy of 53.6%. The remaining samples were misclassified as follows: 1 as a brittle sample incorrectly labelled as elastic; 2 as semi-brittle samples misclassified as brittle and elastic, respectively; 1 as an elastic sample labelled as brittle; 2 as elastic samples as semi-brittle; 3 as elastic samples as highly elastic; and 1 as an additional elastic sample also misclassified as highly elastic. In contrast, the other four models—SVR, Random Forest Regression, Gradient Boosting, and XG-Boost—demonstrated significantly higher classification accuracies, each achieving 85.7%, as depicted in Fig. 20b–e. These models successfully identified 6 as brittle, 7 as semi-brittle, 5 as elastic, and 6 as highly elastic samples correctly. A few misclassifications were noted: one semi-brittle sample was wrongly classified as brittle, another as elastic, and one elastic sample was incorrectly labelled as highly elastic. In summary, these classification results highlight the superior predictive capability of tree-based ensemble models and SVR over Linear Regression for categorizing material behavior based on Young’s modulus. Accurate classification is essential for evaluating the structural response of materials under mechanical loads, particularly considering variations in material composition and processing conditions.

#### Hardness

**Fig. 21:**
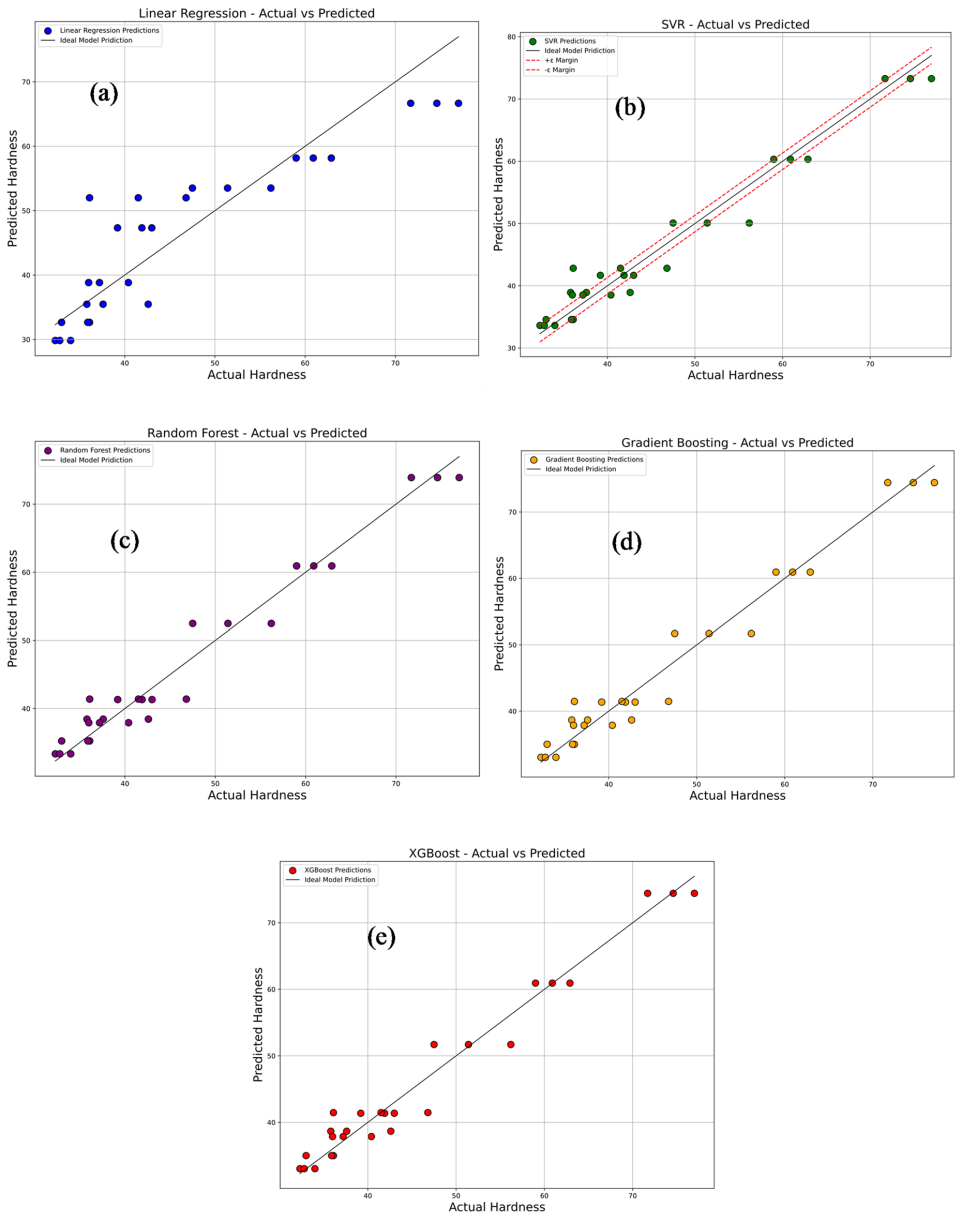
Comparison of actual vs. predicted hardness using machine learning models: (**a**) linear regression; (**b**) support vector regression (SVR); (**c**) random forest; (**d**) gradient boosting; (**e**) XG-Boost.

Figure 21a–e compares the predictions of several machine learning models with the experimentally measured hardness values. The models evaluated include Linear Regression, Support Vector Regression (SVR), Random Forest Regression, Gradient Boosting, and Extreme Gradient Boosting (XG-Boost). In each plot, the predicted values are represented by colored dots plotted against a diagonal reference line, which represents perfect prediction accuracy. As shown in Fig. 21a, the Linear Regression model (blue dots) displays considerable deviation from the reference line, reflecting its limited ability to model the non-linear relationships inherent in the dataset. This limitation is evident from its moderate performance metrics, with an R^2^ of 0.8088, MSE of 33.35, RMSE of 5.775, MAE of 4.5956, and MAPE of 10.3%. The SVR model, illustrated in Fig. 21b with green dots, shows a marked improvement, with predictions more closely aligned to the reference line, though some dispersion persists. It achieves a higher R^2^ of 0.9598, with substantially reduced error values: MSE of 7.004, RMSE of 2.6466, MAE of 2.1462, and MAPE of 4.87%. Further enhancement is observed in the Random Forest model, shown in Fig. 21c with purple dots, where the predicted values cluster more tightly around the diagonal. This model records an R^2^ of 0.9638, MSE of 6.3173, RMSE of 2.5134, MAE of 1.9873, and MAPE of 4.4988%, indicating strong predictive accuracy. The Gradient Boosting model, represented by orange dots in Fig. 21d, delivers even better performance, with predictions closely following the reference line. It achieves an R^2^ of 0.9645, MSE of 6.1958, RMSE of 2.4891, MAE of 1.9383, and MAPE of 4.4061%, confirming excellent prediction consistency and generalization capability. Similarly, the XG-Boost model, shown by red dots in Fig. 21e, achieves nearly identical performance to Gradient Boosting, with its predictions almost perfectly overlapping the reference line. Its comparable R^2^ and error metrics further affirm its accuracy. Overall, these results clearly demonstrate that tree-based ensemble models—particularly Gradient Boosting and XG-Boost—offer superior predictive performance, effectively capturing the complex non-linear patterns in the hardness data. This underlines their suitability for reliably forecasting mechanical properties in materials research.

**Fig. 22:**
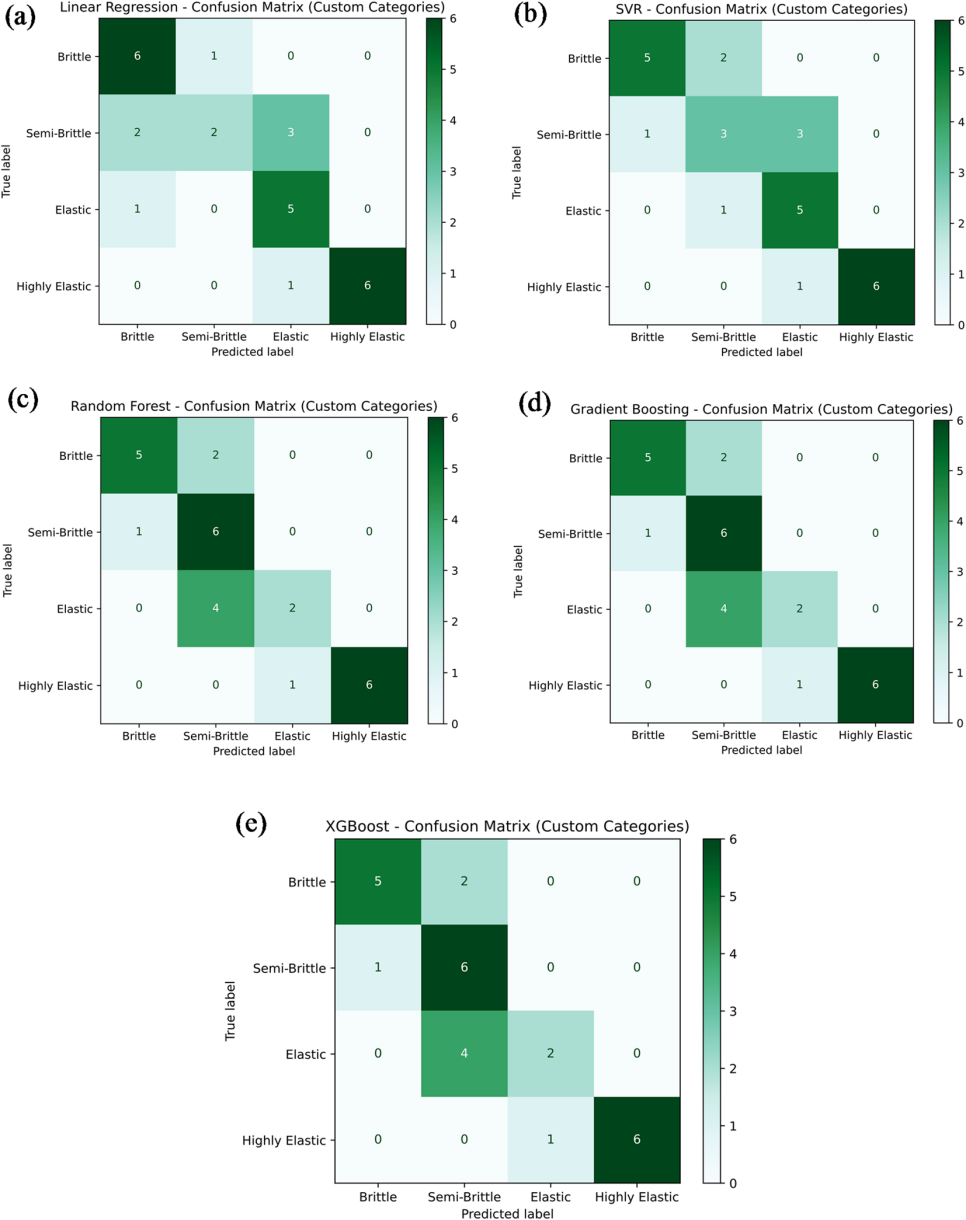
Confusion matrix for machine learning model predicting hardness by (**a**) linear regression; (**b**) support vector regression (SVR); (**c**) random forest; (**d**) gradient boosting; (**e**) XG-Boost.

Figure 22a–e presents the classification performance of various machine learning models in categorizing material hardness into four categories: brittle, semi-brittle, elastic, and highly elastic. The models assessed include Linear Regression, Support Vector Regression (SVR), Random Forest Regression, Gradient Boosting, and Extreme Gradient Boosting (XG-Boost). As illustrated in Fig. 22a, the Linear Regression model achieved a classification accuracy of 53.6%, correctly identifying 6 as brittle, 2 as semi-brittle, 5 as elastic, and 6 as highly elastic samples. The model’s misclassifications comprised 1 as brittle sample incorrectly labelled as semi-brittle; 2 as semi-brittle samples as brittle; 3 as semi-brittle samples as elastic; 1 as elastic sample as brittle; and 1 as highly elastic sample misclassified as elastic. In comparison, the SVR model shown in Fig. 22b demonstrated improved performance, attaining an accuracy of 85.7%. It accurately classified 5 as brittle, 3 as semi-brittle, 5 as elastic, and 6 as highly elastic samples. The remaining misclassifications included 2 as brittle samples as semi-brittle; 1 as semi-brittle sample as brittle; 3 as semi-brittle samples as elastic; 1 as elastic sample as semi-brittle; and 1 as highly elastic sample as elastic. The Random Forest Regression, Gradient Boosting, and XG-Boost models, presented in Fig. 22c–e, each achieved a classification accuracy of 67.87%. These models correctly identified 5 as brittle, 6 as semi-brittle, 2 as elastic, and 6 as highly elastic samples. However, their misclassifications involved 2 as brittle samples as semi-brittle, 1 as semi-brittle sample as brittle, 4 as elastic samples misclassified as semi-brittle, and 1 as highly elastic sample as elastic. In summary, these figures highlight the comparative classification performance of the different machine learning models in predicting material hardness categories. Notably, the SVR model outperformed the others in terms of classification accuracy, while the tree-based ensemble models exhibited moderate yet consistent predictive capability, effectively capturing the trends in material hardness behavior across the defined categories.

**Fig. 23:**
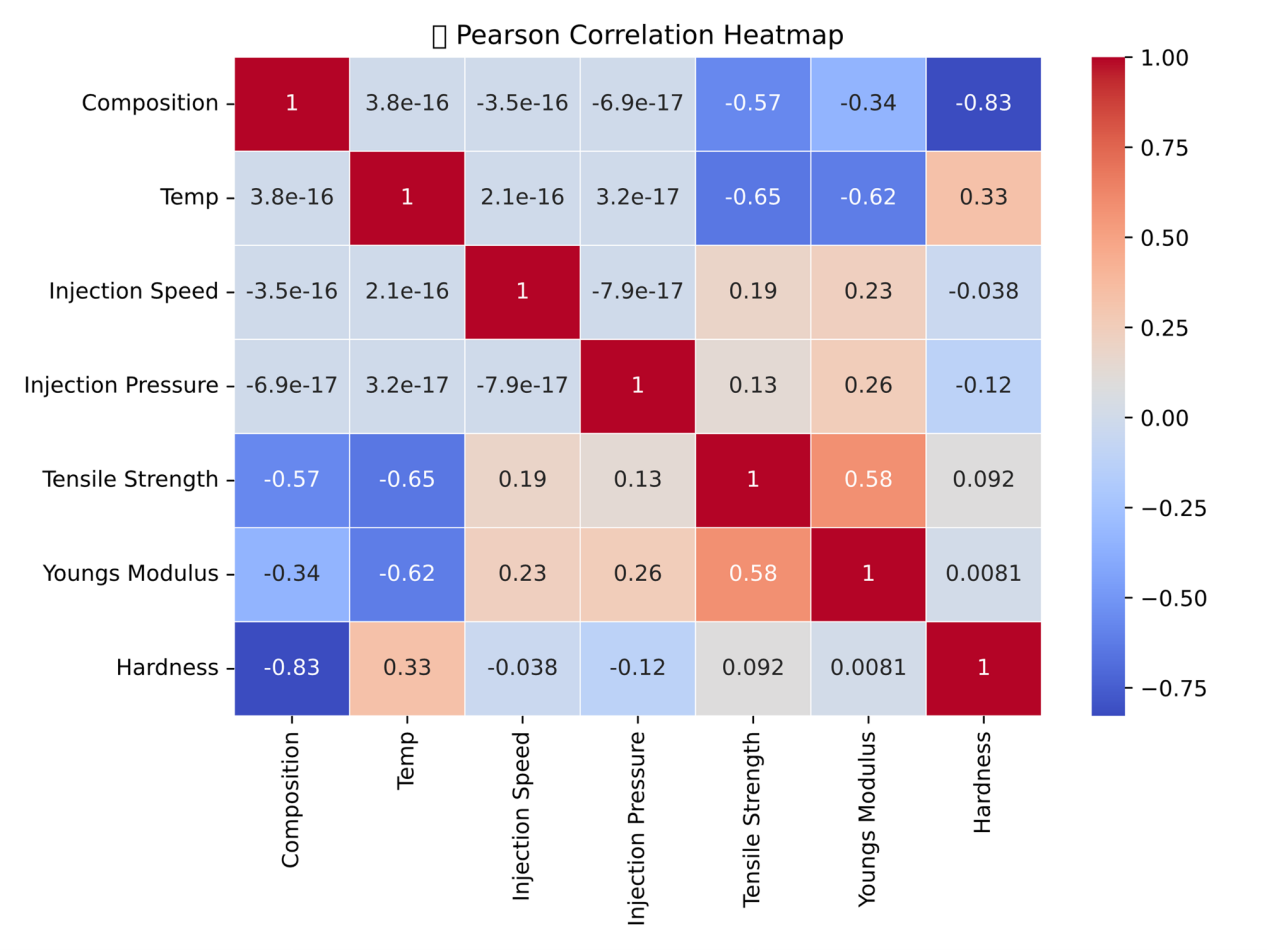
Correlation map between process parameters and mechanical property.

Figure 23 illustrates the influence of injection molding processing parameters on the mechanical properties—Tensile Strength, Young’s Modulus, and Hardness—through the analysis of Pearson correlation coefficients. The heatmap reveals a strong negative correlation between Composition and Hardness (− 0.83) and a moderate negative correlation with Tensile Strength (− 0.57). This suggests that increasing the proportion of certain compositional elements, such as fillers or additives, tends to produce softer materials with diminished tensile strength. Similarly, Temperature exhibits a notable negative correlation with both Tensile Strength (− 0.65) and Young’s Modulus (− 0.62), indicating that higher processing temperatures can adversely affect the mechanical performance of the material. This reduction in strength and stiffness may be attributed to thermal degradation or alterations in molecular alignment during the cooling phase. In contrast, a moderate positive correlation (0.58) is observed between Tensile Strength and Young’s Modulus, which is consistent with typical mechanical behavior where materials with higher tensile strength generally exhibit greater stiffness. Interestingly, both Injection Speed and Injection Pressure show only weak correlations with the mechanical properties, implying that their effects are either less significant or potentially nonlinear when compared to the dominant influences of Composition and Temperature. Overall, this analysis underscores the importance of carefully optimizing both the material composition and processing temperature to achieve desirable mechanical performance in injection-molded components.

#### Cross-validation of ML

To evaluate the robustness and generalization capability of the machine learning models, a k-fold cross-validation (CV) approach was adopted, where the dataset was divided into five subsets and each was used once as a test set while the remaining four served for training. The comparison between CV and train–test R^2^ values showed strong consistency with limited deviation. For tensile strength, SVR, Gradient Boosting, and XGBoost attained CV R^2^ values of 0.936–0.941, compared to train–test values near 0.988, resulting in an error margin of ~ 4.7–5.2%. Hardness predictions yielded CV R^2^ values of 0.879–0.885, closely matching train–test values of 0.954–0.965, with an error of ~ 0.07–0.09. Young’s modulus displayed slightly higher variation, with CV R^2^ of 0.791–0.827 against train–test values of 0.956–0.963, producing an error of ~ 0.13–0.14. Overall, Gradient Boosting and XGBoost provided the best performance, confirming reliable prediction approximately 5% for tensile strength, under 10% for hardness, and 13% for Young’s modulus, confirming their robustness for modelling PLA/CCB composites.

**Fig. 24:**
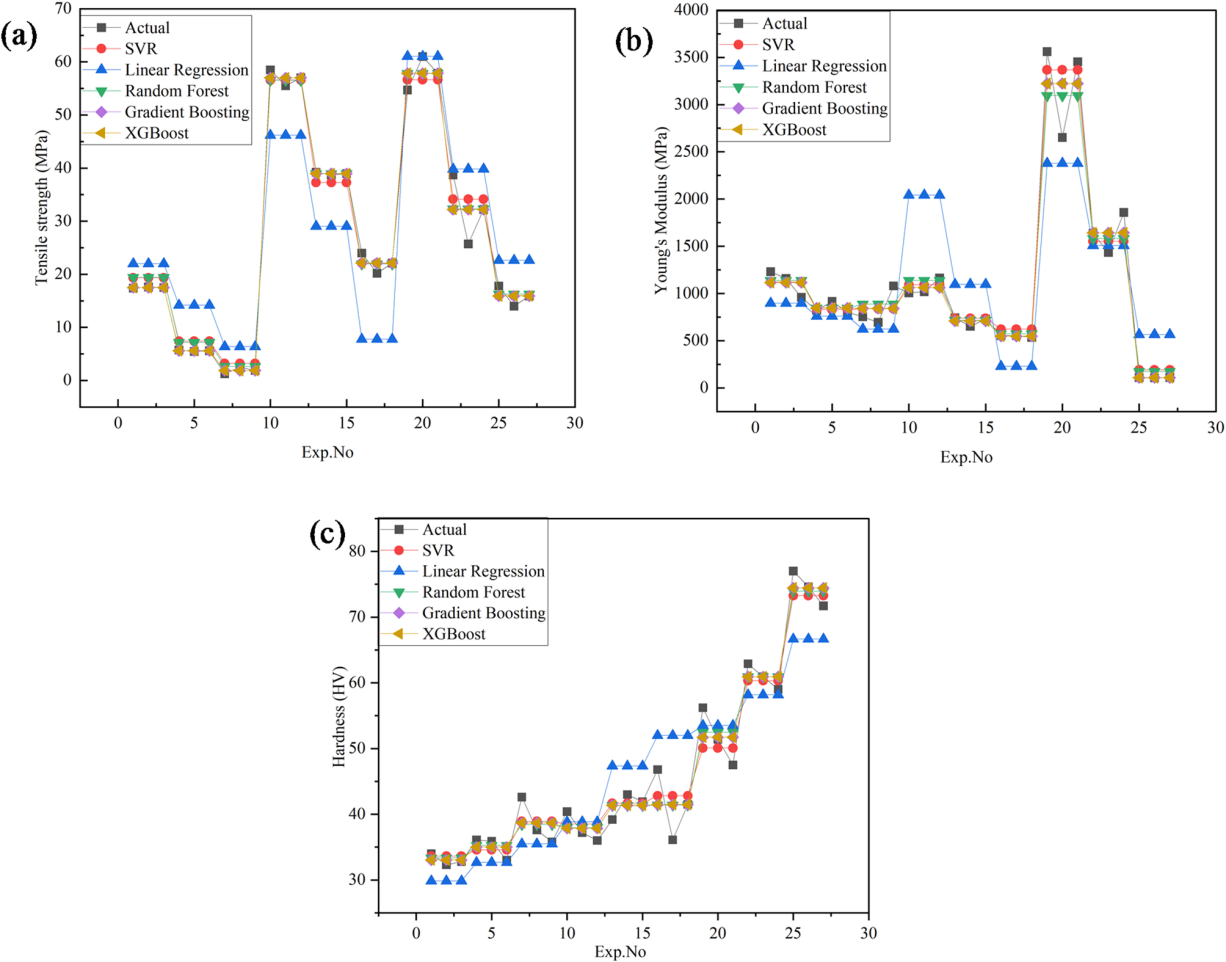
Comparison of experimental results with ML models (**a**) tensile strength; (**b**) Young’s modulus; (**c**) hardness.

Figure 24a compares the experimentally measured and predicted values of tensile strength across 27 experimental runs (Exp. No. 1–27). Predictions were generated using Linear Regression, Support Vector Regression (SVR), Random Forest, Gradient Boosting, and Extreme Gradient Boosting (XG-Boost). Among these, Gradient Boosting and XG-Boost provided the closest match to the experimental data, particularly in Exp. No. 1–3, 10–12, and 22–24, where the predicted values nearly overlapped with the actual measurements. Figure 24b presents the results for Young’s modulus under the same modeling approaches. Similar to tensile strength, Gradient Boosting and XG-Boost achieved the highest accuracy, showing strong alignment with the experimental results in Exp. No. 1–3, 10–12, and 22–24. These models demonstrated minimal deviation, underscoring their capability to capture the underlying mechanical behavior with high reliability.

Figure 24c illustrates the comparison for hardness. While all models produced reasonable predictions, Gradient Boosting and XG-Boost again outperformed the others, especially in Exp. No. 18–27, where near-perfect agreement between predicted and measured values was observed. This consistent performance highlights the robustness of these models in capturing complex nonlinear patterns across different mechanical properties.

## Conclusion

This study demonstrates significant enhancement in the mechanical properties of polylactic acid (PLA) composites reinforced with coconut shell biochar (CCB), particularly in terms of tensile strength, Young’s modulus, and hardness. The experimental results were comprehensively analyzed using ANOVA and further predicted through advanced machine learning models. The key outcomes of the research are summarized below:

- The implementation of a Design of Experiments (DOE) approach using a Taguchi L27 orthogonal array effectively reduced the number of experimental runs while accurately capturing the influence of critical processing parameters.
- Tensile strength and Young’s modulus increased with higher CCB content and lower injection temperatures, while hardness improved with increasing CCB content and higher injection temperatures.
- ANOVA results identified composition as the most influential factor, contributing 50.42% to tensile strength, 38.58% to Young’s modulus, and 78.3% to hardness. Injection temperature also emerged as a significant parameter affecting mechanical performance.
- Among the machine learning models applied, Gradient Boosting and XG-Boost achieved the highest prediction accuracy, with R^2^ values of 98.77% for tensile strength, 96.28% for Young’s modulus, and 96.45% for hardness, confirming their effectiveness in capturing complex material-property relationships.
- Gradient Boosting and XGBoost demonstrated strong generalisation with cross-validation R^2^ values close to train-test values, maintaining prediction errors within approximately 5% for tensile strength, under 10% for hardness, and around 13% for Young’s modulus, confirming their robustness for modelling PLA/CCB composites.

This study optimizes injection molding parameters and PLA/CCB bio-composites using hybrid methodologies to enhance mechanical properties. The work is limited to a narrow filler range and mechanical characterization, restricting a full understanding of multifunctional behavior. Future studies will explore higher filler loadings, thermal, tribological, and durability properties for a comprehensive assessment. The framework will also be extended to 3D printing with advanced hybrid optimization approaches such as GRA, GA–ANN, and DFA^50,51^. Incorporating biofiber^52,53^, biochar–fiber hybrids^54,55^, and novel lignocellulosic fibers^56–58^ can further improve thermal stability, structural integrity, and sustainability of PLA composites^59,60^. These advancements position PLA/CCB composites as promising candidates for lightweight packaging, biomedical devices, consumer electronics, and automotive applications.

## Data Availability

Data is provided within the manuscript.

## Abbreviations

CCB: Coconut shell biochar
PLA: Polylactic acid
wt%: Weight%
ANOVA: Analysis of variance
ML: Machine learning
LR: Linear regression
SVR: Support vector regression
RFR: Random forest regression
GBR: Gradient boosting regression
XGBoost: Extreme gradient boosting
SEM: Scanning electron microscopy
XRD: X-ray diffraction
DOE: Design of experiments
MSE: Mean squared error
RMSE: Root mean squared error
RRMSE: Relative root mean squared error
MAE: Mean absolute error
MAPE: Mean absolute percentage error
R^2^: Coefficient of determination
UTM: Universal testing machine
ASTM: American society for testing and materials
MFI: Melt flow index
PBSA: Poly(butylene succinate-co-adipate)
PHB-HV: Polyhydroxybutyrate-co-valerate
GRA: Grey relational analysis
ANN: Artificial neural network
GA: Genetic algorithm
DFA: Desirability function analysis
HV: Vickers hardness
